# Targeting m^6^A Reader YTHDF1 Enhances Antitumor Immunity and Potentiates Anti‐PD‐L1 Efficacy in Intrahepatic Cholangiocarcinoma

**DOI:** 10.1002/advs.202520403

**Published:** 2026-04-13

**Authors:** Li Luo, Ziqin Liu, Zimin Song, Dong Zhang, Cong Liang, Fei Fang, Kai Lei, Lina Wang, Weikang Chen, Shunli Shen, Ming Kuang, Xiaoxing Li, Jun Yu, Shiyan Wang, Lixia Xu

**Affiliations:** ^1^ Department of Oncology the First Affiliated Hospital Sun Yat‐sen University Guangzhou Guangdong Province China; ^2^ Center of Hepato‐Pancreato‐Biliary Surgery The First Affiliated Hospital Sun Yat‐sen University Guangzhou Guangdong Province China; ^3^ Institute of Precision Medicine The First Affiliated Hospital Sun Yat‐sen University Guangzhou Guangdong Province China; ^4^ Department of Gastrointestinal Surgery The First Affiliated Hospital Sun Yat‐sen University Guangzhou Guangdong Province China; ^5^ Department of Medicine and Therapeutics Institute of Digestive Disease State Key Laboratory of Digestive Disease The Chinese University of Hong Kong Hong Kong SAR China

**Keywords:** FOSL2, immunotherapy, intrahepatic cholangiocarcinoma, MDSCs, YTHDF1

## Abstract

The N6‐methyladenosine (m^6^A) reader YTH N6‐methyladenosine RNA binding protein 1 (YTHDF1) plays a critical role in the tumorigenesis of intrahepatic cholangiocarcinoma (ICC), but its function in the tumor immune microenvironment remains unclear. RNA sequencing analysis of human ICC samples revealed that, among m^6^A‐related regulators, YTHDF1 exhibited the most significant negative correlation with immune score. In multiple ICC mouse models, Ythdf1 overexpression enhanced the recruitment of myeloid‐derived suppressor cells (MDSCs) and suppressed cytotoxic CD8^+^ T cell responses, promoting ICC progression. Immunostaining of human ICC tissue microarray verified that high YTHDF1 protein expression was significantly associated with increased accumulation of MDSCs and decreased infiltration of CD8^+^ T cells. Mechanistically, YTHDF1 bound to the m^6^A site of *FOSL2* mRNA and promoted the translation of FOSL2, a transcription factor driving cytokine CXCL6 expression. Consequently, elevated CXCL6 recruited and activated MDSCs by binding to its receptor CXCR2, leading to the dysfunction of CD8^+^ T cells in ICCs. In addition, targeting YTHDF1 alongside blockade of its downstream chemokine pathway enhanced the efficacy of anti‐PD‐L1 treatment in preclinical ICC mouse models, serving a promising strategy for improving immunotherapy efficacy in ICC.

## Graphical abstract

The m^6^A reader YTHDF1 drives intrahepatic cholangiocarcinoma (ICC) progression by remodeling the tumor immune microenvironment. YTHDF1 promotes MDSC recruitment via activation of m^6^A‐FOSL2‐CXCL6/CXCR2 axis, thereby suppressing CD8^+^ T cell infiltration and function. Targeting YTHDF1 and its downstream chemokine pathway sensitizes ICC to anti‐PD‐L1 therapy, offering a potent strategy to overcome immunotherapeutic resistance.

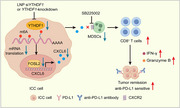

## 1 Introduction

Intrahepatic cholangiocarcinoma (ICC) is a highly lethal malignancy and represents the second most common primary liver cancer, accounting for up to 20% of primary hepatic malignancies [1]. ICC is generally asymptomatic at the early stage, and 70%–80% of patients with ICC are initially diagnosed with advanced disease, ineligible for resection [2]. For the unresectable ICC patients, chemotherapy used to be the standard of care by National Comprehensive Cancer Network (NCCN) guideline. Since 2022, durvalumab (anti‐PD‐L1) plus gemcitabine and cisplatin, a combination treatment of chemotherapy and immunotherapy, has been recommended as the first‐line treatment by the NCCN guideline based on the positive results of the TOPAZ‐1 trial [NCCN2022] [3, 4]. However, the median overall survival was 12.9 versus 11.3 months for participants in the durvalumab plus gemcitabine‐cisplatin group *vs* the placebo plus gemcitabine‐cisplatin group [3, 5, 6], highlighting an urgent need for the identification of potential effective therapeutic targets to enhance patients’ response to immunotherapy.

RNA N^6^‐methyladenosine (m^6^A) is the most abundant internal RNA modification in eukaryotic mRNA and plays a critical role in post‐transcriptional regulation [7]. m^6^A is interpreted by m^6^A readers, thereby regulating RNA stability, splicing, transport, and protein translation [8]. Multiple RNA m^6^A modification modulators have been reported to affect tumor immune microenvironment (TIME) [9]. YT521‐B homology (YTH) m^6^A RNA‐binding protein (YTHDF1) is one of the most important translation‐facilitating m^6^A readers that recruits translation machinery to its target mRNA [10, 11]. By interacting with translation machinery, YTHDF1 increases translation efficiency and promotes protein production from dynamic transcripts that are marked with m^6^A modification [12]. Our previous study has revealed that the expression of YTHDF1 was elevated in ICC tissues compared with adjacent normal tissues. The overexpression of YTHDF1 promoted ICC cell proliferation and cancer tumorigenesis by regulating the translation of epidermal growth factor receptor (EGFR) mRNA in mice and is associated with poor prognosis in ICC patients [13]. However, whether YTHDF1 influences the TIME of ICC remains unknown.

In this study, we reveal that YTHDF1 contributes to the accumulation of myeloid‐derived suppressor cells (MDSCs) and impairs CD8^+^ T cells' function in the TIME of ICC. Through integrative multi‐omics sequencing, we delineate a novel regulatory axis, YTHDF1‐m^6^A‐FOSL2‐CXCL6, through which YTHDF1 drives immunosuppression by recruiting MDSCs, thereby attenuating CD8^+^ T cells' activity. Moreover, we demonstrate that combined blockade of YTHDF1 and CXCR2 significantly augments the efficacy of anti‐PD‐L1 therapy in ICC, supporting a promising therapeutic regimen to potentiate immunotherapy in ICC.

## 2 Results

### 2.1 YTHDF1 Drives ICC Tumorigenesis and Remodels an Immunosuppressive Tumor Microenvironment

**FIGURE 1:**
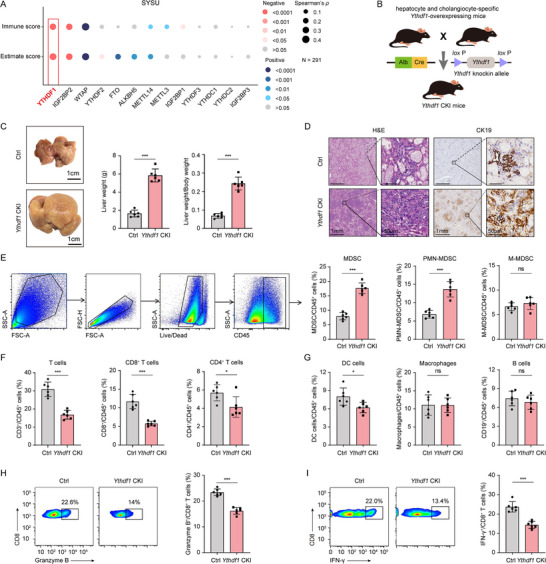
*Ythdf1* overexpression promotes ICC tumorigenesis and inhibits antitumor immunity in *Ythdf1* CKI mice. (A) Immune and Estimate score among the m^6^A regulators in human ICC tumor samples of SYSU cohort with RNA sequencing (*n* = 291). (B) Scheme diagram for the generation of hepatocyte‐specific *Ythdf1*‐overexpressing transgenic mice. (C) Representative gross liver pathological photographs (left), liver weight, and liver weight to body weight ratio (right) in Ctrl and *Ythdf1* CKI mice (*n* = 6). (D) Representative H&E (left) and CK19 IHC staining (right) in Ctrl and *Ythdf1* CKI mice livers (*n* = 6). (E) Gating strategy and Flow cytometry analysis of the percentage of total MDSCs, PMN‐MDSC, and M‐MDSC within CD45^+^ cells in Ctrl and *Ythdf1* CKI ICC tumors (*n* = 6). (F) Flow cytometry analysis of the percentage of CD3^+^ T cells, CD8^+^ T cells, and CD4^+^ T cells within CD45^+^ cells in Ctrl and *Ythdf1* CKI ICC tumors (*n *= 6). (G) Flow cytometry analysis of DC cells, macrophages, and B cells within CD45^+^ cells in Ctrl and *Ythdf1* CKI ICC tumors (*n* = 6). (H,I) Flow cytometry analysis of the percentage of Granzyme B^+^ CD8^+^ T cells (H) and IFN‐γ^+^ CD8^+^ T cells (I) within CD8^+^ cells in Ctrl and *Ythdf1* CKI ICC tumors (*n* = 6). Data are presented as means ± SD, by Student's *t*‐test (C,E–I); ns, no significance; *p < 0.05; **p < 0.01; ***p < 0.001.

By using RNA sequencing (RNA‐seq) data from 291 human intrahepatic cholangiocarcinoma (ICC) tumor samples and 179 matched normal tissues in our internal SYSU cohort, we found that *YTHDF1* mRNA was significantly upregulated in ICC tumors compared to adjacent normal tissues (Figure S1A). Besides, among the main m^6^A regulators (enzymes and readers), we observed strong negative correlations between immune‐related scores and mRNA abundances of YTHDF1 (Figure 1A). To investigate the effect of YTHDF1 on the TIME of ICC, we generated liver cell‐specific *Ythdf1*‐overexpressing transgenic mice (termed as *Ythdf1* CKI) by crossbreeding *lsl*‐*Ythdf1* mice with *Alb*‐cre mice (Figure 1B). The ICC model was established by hydrodynamic tail vein (HTV) injection of two oncogenic plasmids, AKT and YapS127A, along with SB transposase into *Ythdf1* CKI and control mice. Four weeks post‐injection, the mice livers were harvested for evaluation (Figure S1B). *Ythdf1* CKI mice exhibited increased liver tumor burden relative to controls (Figure 1C; Figure S1C). Immunohistochemistry (IHC) staining was used to confirm Ythdf1 expression in ICC tumor tissues (Figure S1D). Hematoxylin‐eosin (H&E) and IHC staining of ICC biomarker cytokeratin 19 (CK19) demonstrated a higher percentage of ICC lesions in *Ythdf1* CKI mice compared with the control mice (Figure 1D). The proliferation rate was significantly increased in *Ythdf1* CKI ICC tumors as assessed by Ki‐67 staining (Figure S1E). Therefore, YTHDF1 accelerated ICC tumorigenesis in *Ythdf1* CKI mouse model.

To characterize the immune landscape of ICC, we performed flow cytometry analysis on ICC tumor tissues. A considerable increase in the infiltration of MDSCs was observed in the *Ythdf1* CKI group compared to the control group. MDSCs are a heterogeneous population of immunosuppressive cells [14], comprising two major subsets: monocytic (M‐MDSC) and polymorphonuclear (PMN‐MDSC) [15]. Specifically, a marked increase in the proportion of PMN‐MDSCs was observed in *Ythdf1* CKI ICC tumors, whereas the frequency of M‐MDSCs remained largely unaffected (Figure 1E). Furthermore, we assessed T cell infiltration in ICC tumors. The percentage of CD3^+^ T cells was significantly reduced in the *Ythdf1* CKI group compared to controls. Notably, tumor‐infiltrating CD8^+^ T cells exhibited a more pronounced reduction than CD4^+^ T cells (Figure 1F). While a slight decrease in dendritic cells was observed, the frequencies of macrophages and B cells remained largely unchanged in *Ythdf1* CKI mice (Figure 1G), collectively indicating a suppressive microenvironment. Critically, the proportions of functional CD8^+^ T subsets, specifically Granzyme B^+^ and IFN‐γ^+^ CD8^+^ T cells, were also significantly diminished (Figure 1H,I). These findings were further corroborated by IHC staining, which confirmed both the increase in PMN‐MDSCs (Figure S1F) and the decrease in functional CD8^+^ T cells (Figure S1G–I). Together, these results demonstrate that hepatocyte‐specific overexpression of Ythdf1 fosters an immunosuppressive TIME in ICC by promoting MDSC infiltration while suppressing functional CD8^+^ T cell accumulation.

### 2.2 YTHDF1 Induces Immunosuppressive TIME in ICC Mouse Models

**FIGURE 2:**
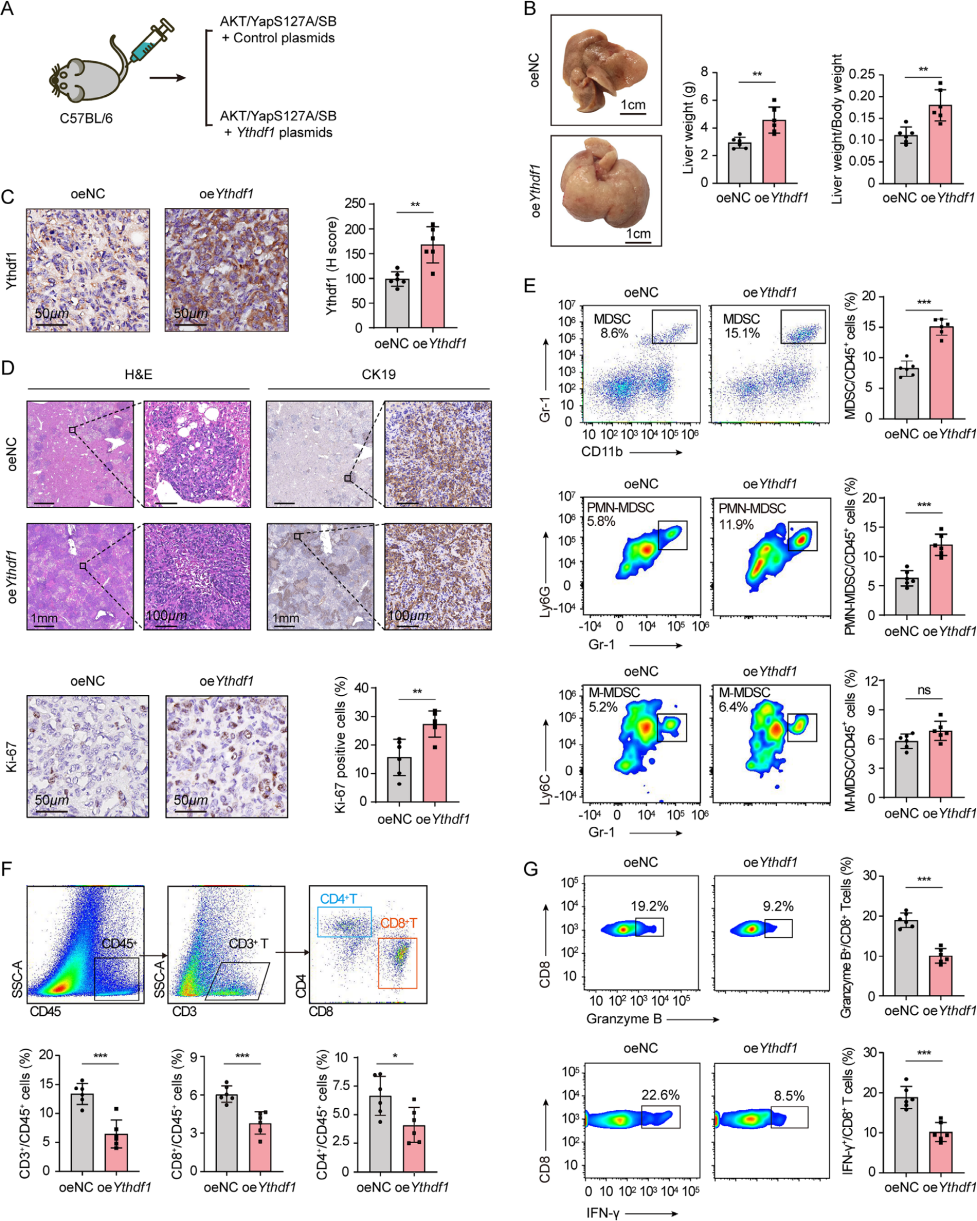
*Ythdf1* overexpression promotes MDSC recruitment and suppresses cytotoxic CD8^+^ T cell responses in hydrodynamic ICC mice models. (A) Schematic diagram of establishing orthotopic *Ythdf1* overexpressing and control ICC mouse model via tail vein hydrodynamic transfection of oncogenic plasmids. (B) Representative liver images (left) and statistical analysis (right) of liver weight and liver weight to body weight ratio in oeNC and oe*Ythdf1* mice (*n* = 6). (C) Representative IHC staining (left) and statistical analysis (right) of Ythdf1 in oeNC and oe*Ythdf1* hydrodynamic ICC mice livers (*n* = 6). (D) Representative H&E staining (upper), IHC staining of CK19 (upper) and Ki‐67 (lower) in oeNC and oe*Ythdf1* hydrodynamic ICC mice livers (*n* = 6). (E) Flow cytometric analysis of MDSC (upper), PMN‐MDSC (median), and M‐MDSC (lower) infiltration in oeNC and oe*Ythdf1* ICC tumors (*n* = 6). (F) Gating strategies for flow cytometry, and quantification of CD3^+^, CD8^+^, and CD4^+^ T cells among CD45^+^ cells in oeNC and oe*Ythdf1* tumors (*n* = 6). (G) Flow cytometry analysis of Granzyme B^+^ (upper) and IFN‐γ^+^ CD8^+^ (lower) T cells within CD8^+^ cells in oeNC and oe*Ythdf1* tumors (*n* = 6). Data are presented as means ± SD, by Student's *t*‐test (B–G); *p < 0.05; **p < 0.01; ***p < 0.001.

We next established an ICC mouse model via hydrodynamic injection of *Ythdf1*‐overexpressing (OE) plasmid or a control plasmid, in combination with Akt, YapS127A, and SB plasmids (Figure 2A). Consistent with observations in *Ythdf1* CKI mice, the tumor burden was significantly elevated in *Ythdf1*‐OE mice compared with the control mice (Figure 2B; Figure S2A). Ythdf1 protein expression in ICC tumor tissues was confirmed by IHC staining (Figure 2C). Keeping with the enhanced tumor growth, *Ythdf1*‐OE tumors exhibited significantly higher percentages of proliferating tumor cells (Figure 2D), increased proportions of PMN‐MDSCs and decreased proportions of total and functional CD8^+^ T cells (Figure 2E–G; Figure S2B–E). These results further substantiate the role of YTHDF1 in promoting an immunosuppressive tumor microenvironment in ICC.

To further validate the impact of YTHDF1 on ICC TIME, we established an orthotopic ICC model in immunocompetent C57BL/6 mice by injecting *Ythdf1*‐knockdown or control mouse ICC LTP‐C9 cells into the liver capsules (Figure S3A,B). Tumor growth of ICC xenografts in the Ythdf1‐knockdown group was significantly dampened, characterized by smaller tumor mass, reduced tumor weight, and liver weight compared with that in control mice (Figure S3C,D). Ythdf1 knockdown efficacy in ICC xenografts was confirmed by IHC staining (Figure S3E). Ki‐67 staining showed reduced proportions of proliferative cells in tumors in the *Ythdf1*‐knockdown group compared with controls (Figure S3F). Consistent with the effects of Ythdf1 overexpression, Ythdf1 knockdown led to a pronounced reduction in PMN‐MDSC infiltration (Figure S4A,B), along with a significant increase in total CD8^+^ T cells and functional cytotoxic CD8^+^ T cells (Granzyme B^+^ CD8^+^ T cells and IFN‐γ^+^ CD8^+^ T cells) in ICC tumors (Figure S4C–F). Taken together, these findings demonstrate that YTHDF1 perturbation reverses the immunosuppressive TIME in ICC.

### 2.3 YTHDF1 Induces PMN‐MDSC Accumulation via CXCL6‐CXCR2 Axis

**FIGURE 3:**
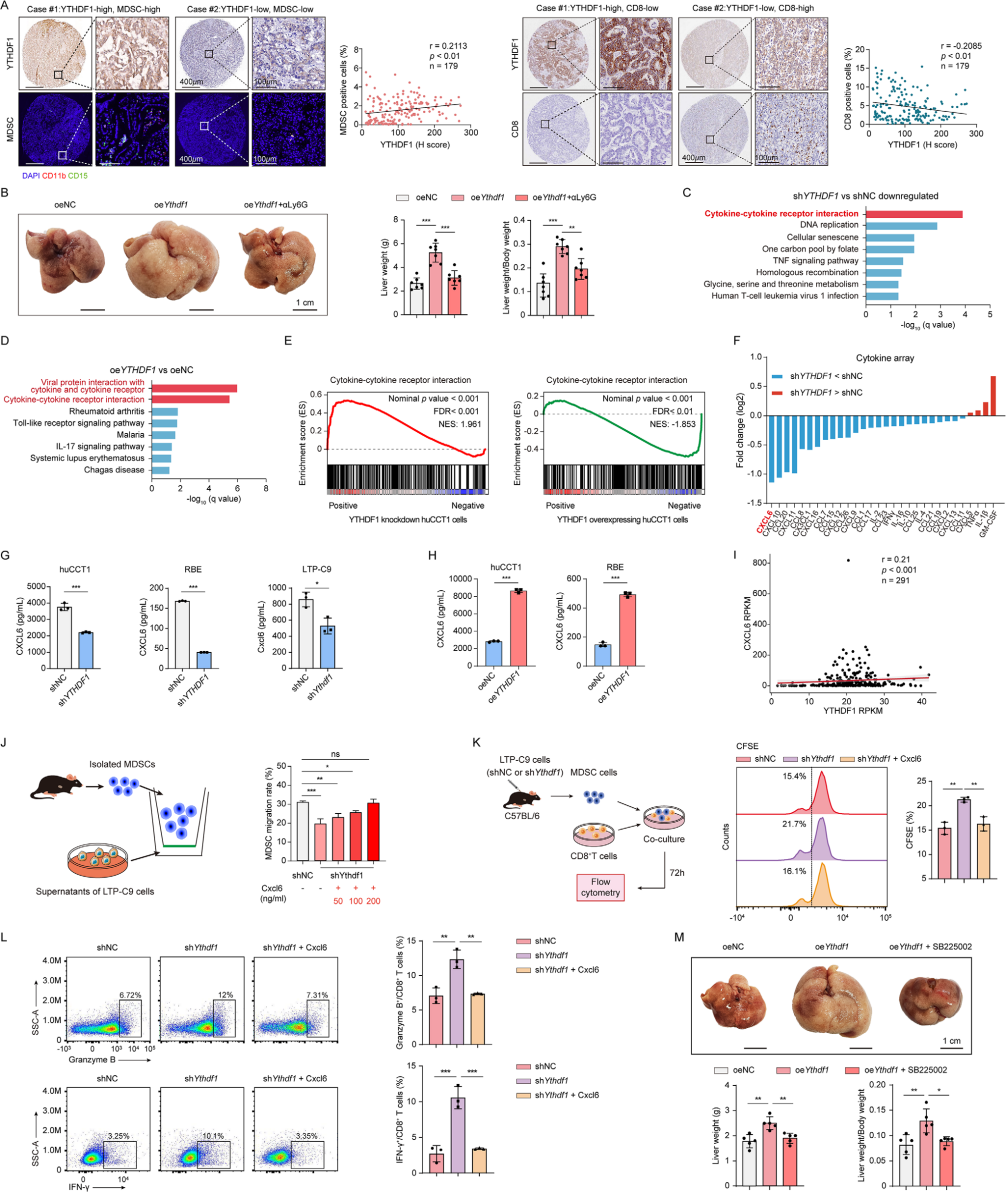
YTHDF1 induces PMN‐MDSC accumulation via the CXCL6‐CXCR2 axis. (A) Correlation analysis between YTHDF1 protein expression and MDSC infiltration (left), as well as YTHDF1 and CD8^+^ T cell infiltration (right) in human ICC tissue microarrays. (B) Representative liver images (left) and quantification (right) of liver weight and liver weight to body weight ratio in oeNC, oe*Ythdf1*, and oe*Ythdf1* with αLy6G‐treated mice (*n* = 7). (C) Kyoto Encyclopedia of Genes and Genomes (KEGG) pathway analysis of differentially expressed genes in *YTHDF1*‐knockdown versus control huCCT1 cells. (D) KEGG pathway analysis of differentially expressed genes in *YTHDF1*‐overexpressing versus control huCCT1 cells. (E) GSEA analysis of cytokine–cytokine receptor interaction pathway in both YTHDF1‐knockdown (left) and overexpressing (right) huCCT1 cells. (F) Quantification of cytokines and chemokines in conditioned medium from *YTHDF1*‐knockdown and control huCCT1 cells using Luminex liquid suspension chip. (G) ELISA analysis of CXCL6 secretion in the conditioned medium of huCCT1, RBE cells, and LTP‐C9 cells following YTHDF1 knockdown (*n* = 3). (H) ELISA analysis of CXCL6 secretion in the conditioned medium of huCCT1, RBE cells following YTHDF1 overexpression (*n* = 3). (I) Correlation analysis between YTHDF1 and CXCL6 mRNA expression in human ICC tumor samples (SYSU cohort) with RNA sequencing (*n* = 291). (J) Schematic diagram of the in vitro MDSC migration assay workflow and the analysis of the MDSC migration assay induced by shNC or sh*Ythdf1* conditioned medium with or without Cxcl6 supplementation. (K) Schematic flow diagram of T cell suppression assay and proliferation of CFSE‐labeled CD8^+^ T cells co‐cultured with MDSCs at a 1:1 ratio for 72 h, assessed by flow cytometry. (L) Flow cytometric analysis of Granzyme B^+^ (upper) and IFN‐γ^+^ (lower) CD8^+^ T cells after co‐cultured with MDSCs for 72 h (*n* = 3). (M) Representative liver images (upper) and quantification (lower) of liver weight and liver weight to body weight ratio in oeNC, oe*Ythdf1*, and oe*Ythdf1* with SB225002‐treated mice (*n* = 5). Data are presented as means ± SD, by Pearson correlation test (A), one‐way Anova (B,J–M), by Spearman correlation test (I), and Student's *t*‐test (G,H); *p < 0.05; **p < 0.01; ***p < 0.001.

Given the expression level of YTHDF1 can affect TIME in ICC in mice models, we also validated the relationship between YTHDF1, MDSCs, and CD8^+^ T cells in human ICC samples. We performed IHC and immunofluorescence (IF) analyses on tissue microarrays comprising 179 ICC samples collected in our center. Results showed that in human ICC samples, YTHDF1 presented a positive correlation with MDSCs (*r* = 0.2113, *p* < 0.01), whereas a negative relationship with CD8^+^ T cells (*r* = −0.2085, *p* < 0.01) (Figure 3A), confirming the association of YTHDF1 expression with MDSCs and CD8^+^ T cells infiltration. To further establish the functional contribution of PMN‐MDSCs to YTHDF1‐mediated ICC progression in vivo, PMN‐MDSCs were depleted using an antibody against Ly6G in *Ythdf1*‐overexpressing tumors in a hydrodynamic injection ICC mouse model. Indeed, PMN‐MDSC blockade greatly abolished the enhanced tumor growth by Ythdf1 overexpression (Figure 3B; Figure S5A). Therefore, YTHDF1 drives PMN‐MDSC accumulation and activation, contributing to the establishment of an immunosuppressive TIME.

To uncover the mechanism by which YTHDF1 promotes immunosuppressive TIME, we generated *YTHDF1*‐knockdown and overexpressing cells in human ICC huCCT1 and RBE cells, which were confirmed by Reverse transcription‐quantitative polymerase chain reaction (RT‐qPCR) and Western blot assays (Figure S6A–D). RNA‐seq was performed in *YTHDF1*‐knockdown and overexpressing huCCT1 cells, along with their respective control cells. Kyoto Encyclopedia of genes and genomes (KEGG) pathway analysis and gene set enrichment analysis (GSEA) showed that “cytokine–cytokine receptor interaction” was prominently enriched in both *YTHDF1*‐knockdown and overexpressing cells (Figure 3C–E; Figure S6E), implicating cytokines as key mediators in YTHDF1's regulation of TIME. We next examined the expression of main cytokines and chemokines in the culture supernatants affected by YTHDF1 using Cytokine multiplex immunoassays. Among the cytokines and chemokines examined, the top four were C‐X‐C motif chemokine ligand 6 (CXCL6), CXCL10, CCL20, and CXCL11, among which CXCL6 exhibited the most pronounced downregulation in *YTHDF1*‐knockdown huCCT1 cells (Figure 3F). To validate the YTHDF1‐mediated regulation of these cytokines and chemokines, we performed ELISA on the top four candidates in *YTHDF1*‐knockdown and overexpressing ICC cells. Consistently, CXCL6 was the only candidate significantly decreased upon YTHDF1 depletion and increased upon its overexpression in both huCCT1 and RBE cells (Figure 3G,H; Figure S6F–K). To further investigate the correlation between YTHDF1 and CXCL6 expression in human ICC tissues, we analyzed their expression levels in both the SYSU ICC cohort (*n* = 291) and the external GSE32225 dataset (*n *= 155). A significant positive correlation between YTHDF1 and CXCL6 expression was consistently observed in both cohorts (SYSU cohort*: r* = 0.21, *p* < 0.001; GSE32225: *r* ^2^  = 0.35, *p* < 0.001) (Figure 3I; Figure S6L).

CXCL6 has been reported as a chemotactic agent that regulates the migration of PMN‐MDSCs into the TIME mainly through binding to its receptor CXCR2 [16, 17, 18]. We thus hypothesized that YTHDF1 regulates the function of MDSCs and/or CD8^+^ T cells by controlling the secretion of CXCL6. Conditioned medium derived from *Ythdf1*‐knockdown LTP‐C9 cells significantly decreased MDSC migration, addition of recombinant Cxcl6 protein significantly rescued the suppressed migration ability of MDSCs in a dose‐dependent manner (Figure 3J). However, no significant differences were observed in the effects of conditioned medium on the proliferation and functions of CD8^+^ T cells isolated from the spleens of wild‐type (WT) C57BL/6 mice between *Ythdf1*‐knockdown LTP‐C9 cells and controls (Figure S7A,B). Since MDSCs suppress CD8^+^ T cell activity, the effects of MDSCs in *Ythdf1*‐knockdown and control ICC tumors on CD8^+^ T cells were evaluated. We isolated MDSCs from *Ythdf1*‐knockdown and control LTP‐C9 liver orthotopic tumors and co‐cultured MDSCs with CD8^+^ T cells isolated from the spleens of WT C57BL/6 mice. As expected, the abilities of MDSCs derived from *Ythdf1*‐knockdown tumors in inhibiting the proliferation and function of CD8^+^ T cells were significantly compromised compared to controls, while Cxcl6 supplementation fully reversed those reduced abilities of MDSCs derived from *Ythdf1*‐knockdown tumors (Figure 3K,L), suggesting a direct role of YTHDF1 in regulating MDSCs rather than CD8^+^ T cells in TIME.

CXCL6 functions by binding to its receptors CXCR2, which is an abundant receptor on the surface of PMN‐MDSCs [19]. Thus, we blocked CXCL6‐CXCR2 signaling in vivo by treating the mice bearing *Ythdf1*‐overexpressing ICC tumors with the selective CXCR2 antagonist SB225002. Blockade of CXCR2 signaling significantly relieved the tumor‐promoting effect by YTHDF1 overexpression (Figure 3M; Figure S7C). Collectively, these data demonstrated that YTHDF1 promoted the accumulation and function of PMN‐MDSCs via the CXCL6‐CXCR2 axis, thereby accelerating ICC progression.

### 2.4 YTHDF1 Promotes FOSL2 Translation to Upregulate CXCL6 in ICC

**FIGURE 4:**
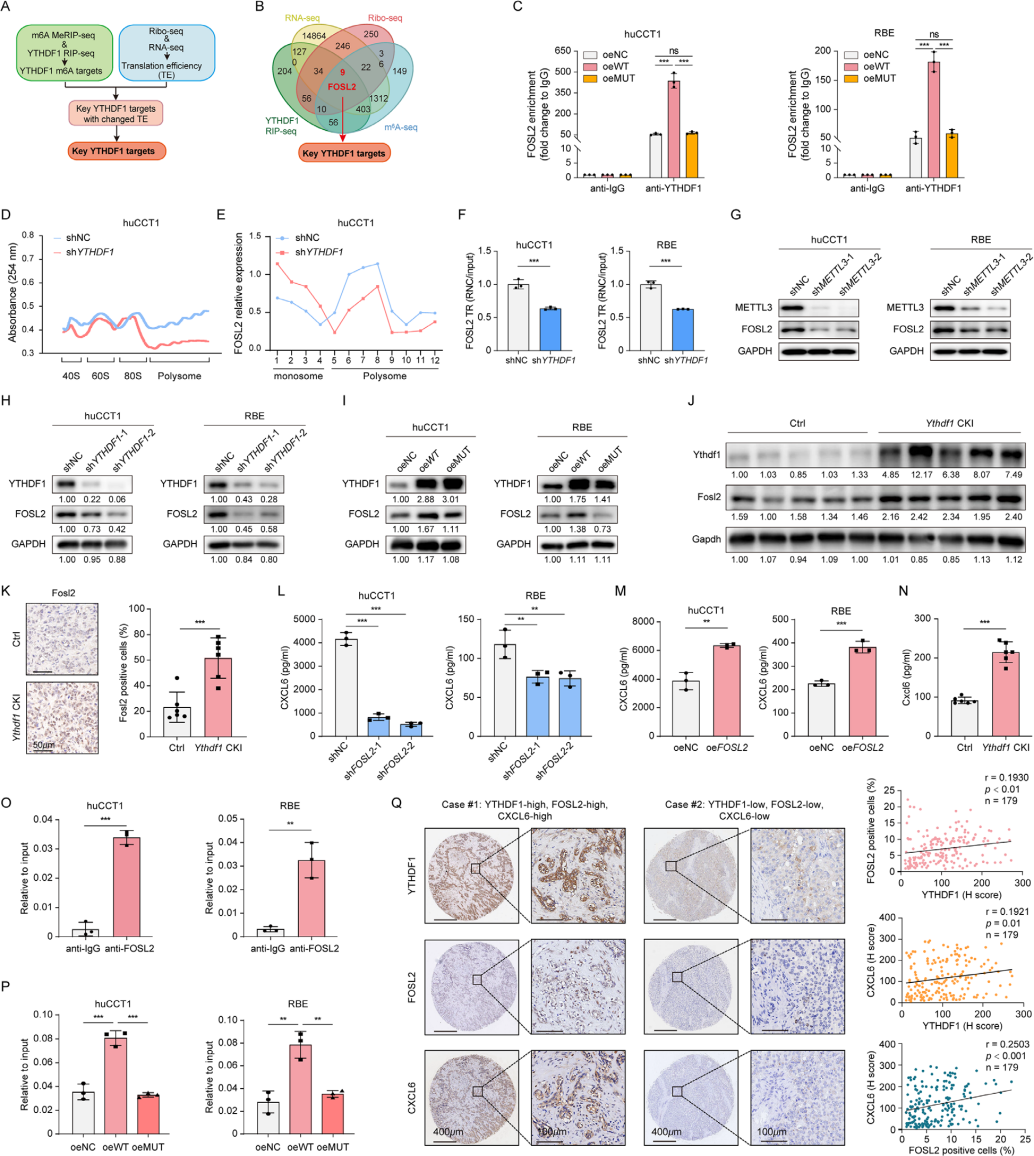
YTHDF1 upregulates CXCL6 through promoting FOSL2 mRNA translation. (A,B) Schematic workflow (A) and Venn diagram (B) illustrating the multiomics strategy used to identify candidate YTHDF1 target genes. (C) RIP‐qPCR analysis showing the binding ability of YTHDF1 with *FOSL2* mRNA in control (oeNC), WT YTHDF1 (oeWT), or YTHDF1 MUT (oeMUT) huCCT1 and RBE cells. (D) Polysome profiling of huCCT1 cells with control (shNC) or YTHDF1 knockdown (sh*YTHDF1*). (E) Polysome‐qPCR assay of huCCT1 shNC and sh*YTHDF1* cells. (F) RNC‐qPCR analysis of *FOSL2* in huCCT1 and RBE shNC and sh*YTHDF1* cells. (G) Western blot showing FOSL2 protein expression level in huCCT1 and RBE shNC and sh*METTL3* cells. (H) Western blot showing FOSL2 protein expression level in huCCT1 and RBE shNC and sh*YTHDF1* cells. (I) Western blot showing FOSL2 protein expression level in huCCT1 cells with overexpression of oeNC, oeWT, and oeMUT. (J) Western blot showing Fosl2 expression in ICC tumors from Ctrl and *Ythdf1* CKI mice. (K) Representative images (left) and quantification (right) of Fosl2 staining in Ctrl and *Ythdf1* CKI ICC tumors. (L) ELISA showing CXCL6 level in conditioned medium of shNC and sh*FOSL2* huCCT1 and RBE cells. (M) ELISA showing CXCL6 level in conditioned medium of oeNC and oe*FOSL2* huCCT1 and RBE cells. (N) ELISA showing Cxcl6 level in ICC tumors from Ctrl and *Ythdf1* CKI mice. (O) ChIP‐qPCR showing direct binding of FOSL2 to the *CXCL6* promoter region in huCCT1 and RBE cells. (P) ChIP‐qPCR assay showing the binding ability of FOSL2 with *CXCL6* promoter in oeNC, oeWT, and oeMUT huCCT1 and RBE cells. (Q) IHC staining showing the correlation between YTHDF1 and FOSL2, YTHDF1 and CXCL6, as well as FOSL2 and CXCL6 in ICC tissue microarrays. Data are presented as means ± SD, by Student's *t*‐test (F,K,M–O), one‐way Anova (C,L,P), and Pearson correlation test (Q); ns, no significance; *p < 0.05; **p < 0.01; ***p < 0.001.

YTHDF1 is a m^6^A reader preferentially binding to m^6^A‐modified mRNAs to facilitate translation [20]. To elucidate the underlying molecular mechanism by which YTHDF1 induces CXCL6 secretion, we applied multiomics sequencing to screen out downstream targets regulated by YTHDF1 by means of m^6^A modification in huCCT1 cells, including RNA‐seq, MeRIP‐seq, YTHDF1 RIP‐seq, and Ribo‐seq (Figure 4A). MeRIP‐seq revealed that m^6^A peaks were mainly located in the coding sequence region (CDS) and 3’‐untranslated region and “RRAC” was the most enriched motif identified in these peaks (Figure S8A). YTHDF1 knockdown makes no difference on the overall m^6^A levels (Figure S8B). Notably, no m^6^A peak was observed in CXCL6, implying that CXCL6 was not a direct target of YTHDF1. To uncover the prospective targets that work in YTHDF1‐induced CXCL6 upregulation, we overlapped the m^6^A MeRIP‐seq and YTHDF1 RIP‐seq data to identify YTHDF1 m^6^A target genes. We intersected genes showing unchanged expression in RNA‐seq with those exhibiting elevated translation efficiency in Ribo‐seq. Nine key YTHDF1 targets were finally determined by integration of the multiomics data (Figure 4A,B). Among the nine genes, only Fos‐like antigen 2 (FOSL2/FRA‐2) was predicted to bind to the CXCL6 promoter region using the JASPAR CORE database (https://jaspar.elixir.no/) [21]. FOSL2 is a protein‐coding gene that belongs to the activator protein 1 (AP‐1) transcription factor family [22]. We thus hypothesized that FOSL2 is the mediator by which YTHDF1 regulates CXCL6 production and MDSC recruitment.

To explore whether YTHDF1 regulates FOSL2 translation in a m^6^A modification‐dependent manner, we constructed a mutant YTHDF1 (YTHDF1‐MUT) with mutations of Lys395 and Tyr397 to alanine (K395A and Y397A), which disrupt its m^6^A‐binding capacity [23, 24]. We generated ICC cells with the overexpression of either WT YTHDF1 or YTHDF1‐MUT. Subsequent RIP‐qPCR analysis showed that the binding ability of YTHDF1 to *FOSL2* mRNA was increased in the YTHDF1‐WT overexpressing cells, whereas this interaction was diminished in YTHDF1‐MUT overexpressing cells (Figure 4C). RT‐qPCR assays indicated that YTHDF1 did not affect FOSL2 mRNA abundance (Figure S8C,D), suggesting that YTHDF1 exerts its regulatory role at the translational level in ICC cells. In support of this, polysome profiling demonstrated a reduced association of *FOSL2* mRNA with ribosomes following YTHDF1 knockdown (Figure 4D,E). Ribosome nascent‐chain complex‐bound mRNA (RNC)‐qPCR further corroborated these findings, showing that YTHDF1 depletion significantly suppressed *FOSL2* translation efficiency in human ICC cells (Figure 4F). To explore the dependency of YTHDF1‐mediated *FOSL2* translation on m^6^A modification, we ablated METTL3, the RNA methyltransferase responsible for m^6^A formation, in ICC cells. This manipulation led to a marked reduction in FOSL2 protein levels (Figure 4G), implicating m^6^A as a critical regulator of FOSL2 expression. Accordingly, in ICC cells, Western blot assay showed that YTHDF1 knockdown reduced FOSL2 levels (Figure 4H), whereas its overexpression increased FOSL2 expression, which was abolished by the YTHDF1‐MUT (Figure 4I). To verify the YTHDF1‐FOSL2‐CXCL6 signaling in vivo, we isolated ICC lesions from *Ythdf1* CKI and control mice. RT‐qPCR and Western blot analysis confirmed that Ythdf1 overexpression did not affect *Fosl2* mRNA levels (Figure S8E), but significantly increased Fosl2 protein expression in *Ythdf1*‐overexpressing ICC tumors (Figure 4J). Consistently, the percentage of Fosl2‐positive cells was also elevated in *Ythdf1* CKI ICC tumors compared to controls (Figure 4K). In addition, we constructed *FOSL2*‐knockdown ICC cells (Figure S8F,G). ELISA analysis of the culture medium from ICC cells revealed that FOSL2 depletion significantly inhibited both the expression and secretion of CXCL6 (Figure 4L; Figure S8H). Conversely, overexpression of FOSL2 upregulated CXCL6 expression in ICC cells (Figure 4M; Figure S8I–K). Consistent with these in vitro findings, ELISA further demonstrated that Cxcl6 concentrations were elevated in *Ythdf1* CKI mice compared to controls (Figure 4N). Mechanistically, Chromatin Immunoprecipitation (ChIP)‐qPCR analysis revealed that FOSL2 protein could directly bind to the *CXCL6* promoter in ICC cells. This interaction was enhanced by YTHDF1‐WT overexpression, but not by the YTHDF1‐MUT (Figure 4O,P), suggesting a m^6^A‐dependent mechanism.

We further validated the YTHDF1‐FOSL2‐CXCL6 signaling axis in human ICC clinical samples. Immunohistochemical analysis of tissue microarrays revealed that YTHDF1 protein expression was positively correlated with FOSL2 (*n* = 179, *r* = 0.1930, *p* < 0.01) and CXCL6 (*n* = 179, *r* = 0.1921, *p* < 0.01). A positive correlation was also observed between FOSL2 and CXCL6 expression (*n *= 179, *r* = 0.2503, *p* < 0.001) (Figure 4Q). These findings proved that YTHDF1 promoted FOSL2 translation, leading to subsequent upregulation of CXCL6. Collectively, our data indicate that YTHDF1 facilitates MDSC recruitment in ICC through the FOSL2‐CXCL6 signaling axis.

### 2.5 YTHDF1‐Knockdown Represses Tumorigenesis and Improves the Efficacy of Anti‐PD‐L1 Treatment in ICC

**FIGURE 5:**
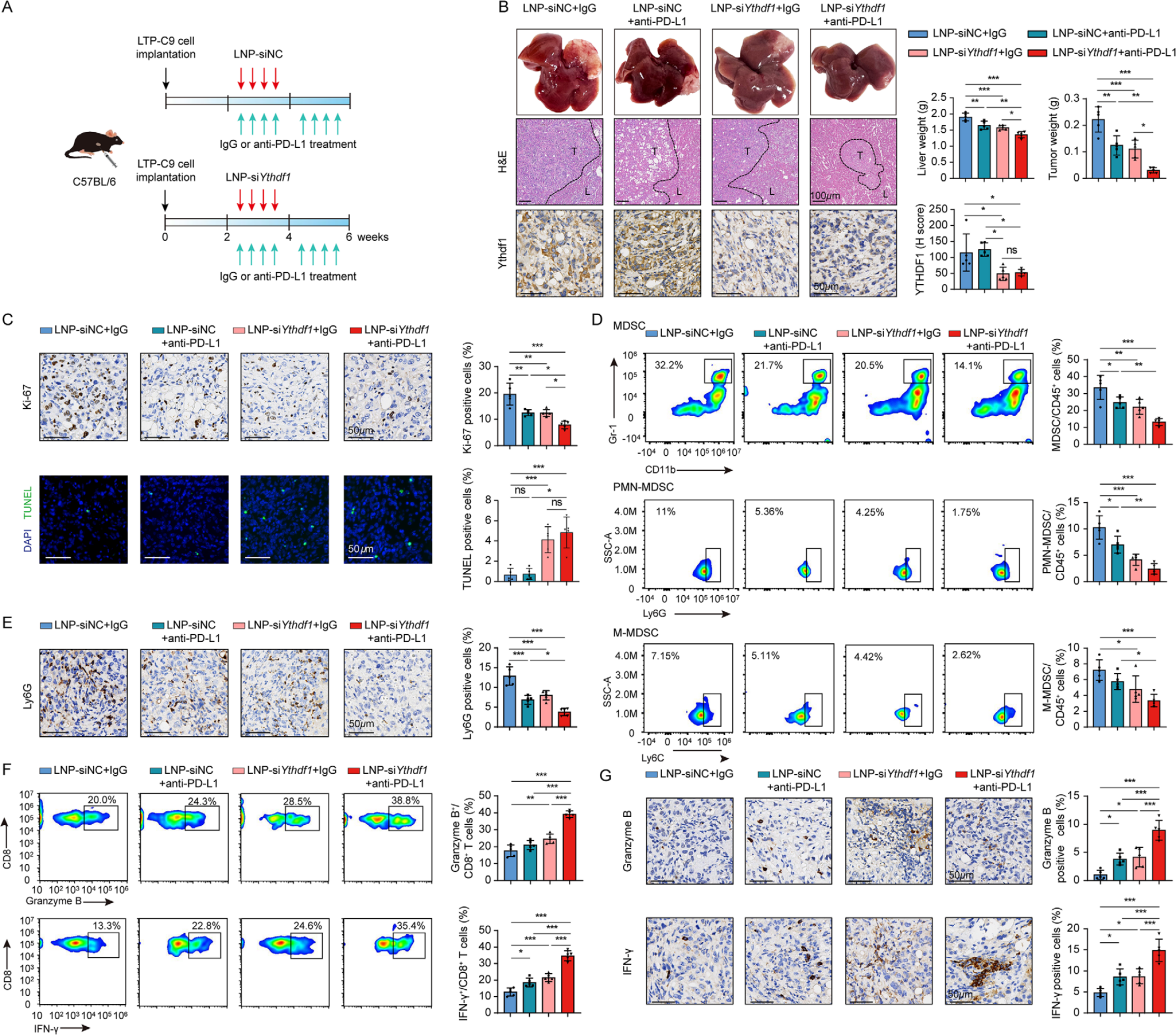
LNP‐si*Ythdf1* reduces tumor burden and improves the efficacy of anti‐PD‐L1 treatment in ICC. (A) Schematic diagram of the orthotopic ICC model established by LTP‐C9 tumor cell orthotopic implantation and the combination therapy of LNP‐si*Ythdf1/LNP‐siNC* and anti‐PD‐L1/IgG. (B) Representative images of H&E, IHC staining (Ythdf1), and statistical analysis of orthotopic ICC tumors with the indicated treatment (*n* = 5). (C) Representative IHC staining, TUNEL staining, and quantification in orthotopic tumors with the indicated treatment (*n* = 5). (D) Flow cytometry analysis of MSDCs (upper), PMN‐MDSCs (median), and M‐MDSCs (lower) in orthotopic tumors with the indicated treatment (*n* = 5). (E) Representative IHC staining and quantification of Ly6G in orthotopic tumors with the indicated treatment (*n* = 5). (F) Flow cytometry analysis of Granzyme B^+^ (upper) and IFN‐γ^+^ (lower) CD8^+^ T cells in orthotopic tumors with the indicated treatment (*n* = 5). (G) Representative IHC staining and quantification of Granzyme B (upper) and IFN‐γ (lower) in orthotopic tumors (*n* = 5). Data are presented as means ± SD, by one‐way Anova (B–G); ns, no significance; *p < 0.05; **p < 0.01; ***p < 0.001.

MDSCs have been regarded to inhibit immunotherapy efficacy in various cancer types [25, 26]. Given that YTHDF1 recruits immunosuppressive MDSCs in ICC, we next sought to examine whether knockdown of YTHDF1 could augment the response to anti‐PD‐L1 therapy. Lipid nanoparticle (LNP)‐formulated siRNA represents a well‐established drug delivery system and a promising therapeutic strategy for precision medicine. Upon intravenous administration, LNP‐siRNA predominantly accumulates in the liver [27, 28]. To investigate the therapeutic potential of targeting YTHDF1, we formulated *Ythdf1*‐targeting and control siRNA into lipid nanoparticles (LNP‐siNC/LNP‐si*Ythdf1*) and evaluated their efficacy in combination with anti‐PD‐L1 therapy in an orthotopic ICC mouse model. Specifically, orthotopic tumors were established using LTP‐C9 cells. Two weeks later, mice were randomized and treated with LNP‐si*Ythdf1*, anti‐PD‐L1, or their combination (Figure 5A). Notably, combined therapy with LNP‐si*Ythdf1* and anti‐PD‐L1 markedly reduced tumor burden compared to the control group (Figure 5B; Figure S9). Pathological analysis confirmed effective Ythdf1 depletion in the combination group, accompanied by decreased tumor cell proliferation, increased apoptosis, as evidenced by Ki‐67 and TUNEL staining (Figure 5B,C). Furthermore, the combination treatment robustly suppressed MDSC recruitment (Figure 5D,E) and enhanced CD8^+^ T cell effector function (Figure 5F,G). Collectively, these findings demonstrate that targeting YTHDF1 potentiates the anti‐tumor efficacy of anti‐PD‐L1 therapy and represents a promising therapeutic strategy for ICC.

Furthermore, we established an orthotopic ICC mouse model using LTP‐C9 cells with stable Ythdf1 knockdown or control, and treated the mice with either IgG or anti‐PD‐L1 therapy (Figure S10A). Compared to either *Ythdf1*‐knockdown or anti‐PD‐L1 treatment, *Ythdf1*‐knockdown combined with anti‐PD‐L1 treatment exerted the most conspicuous suppressive effect on ICC tumor growth (Figure S10B). H&E and IHC analyses validated that tumor burden and cell proliferation were most significantly reduced, whereas apoptosis was increased in the combination group (Figure S10C–F). Flow cytometry and IHC staining further revealed that *Ythdf1*‐knockdown combined with anti‐PD‐L1 therapy significantly reduced MDSC recruitment, particularly PMN‐MDSCs, which were prone to a more obvious decrease (Figure S10G,H). Conversely, the infiltration and functional activity of CD8^+^ T cells were markedly enhanced in the combination group (Figure S10I,J), indicating a more robust anti‐tumor immune response.

### 2.6 Combined Blockade of YTHDF1 and CXCR2 Synergistically Enhances Anti‐PD‐L1 Therapy to Suppress ICC Progression

**FIGURE 6:**
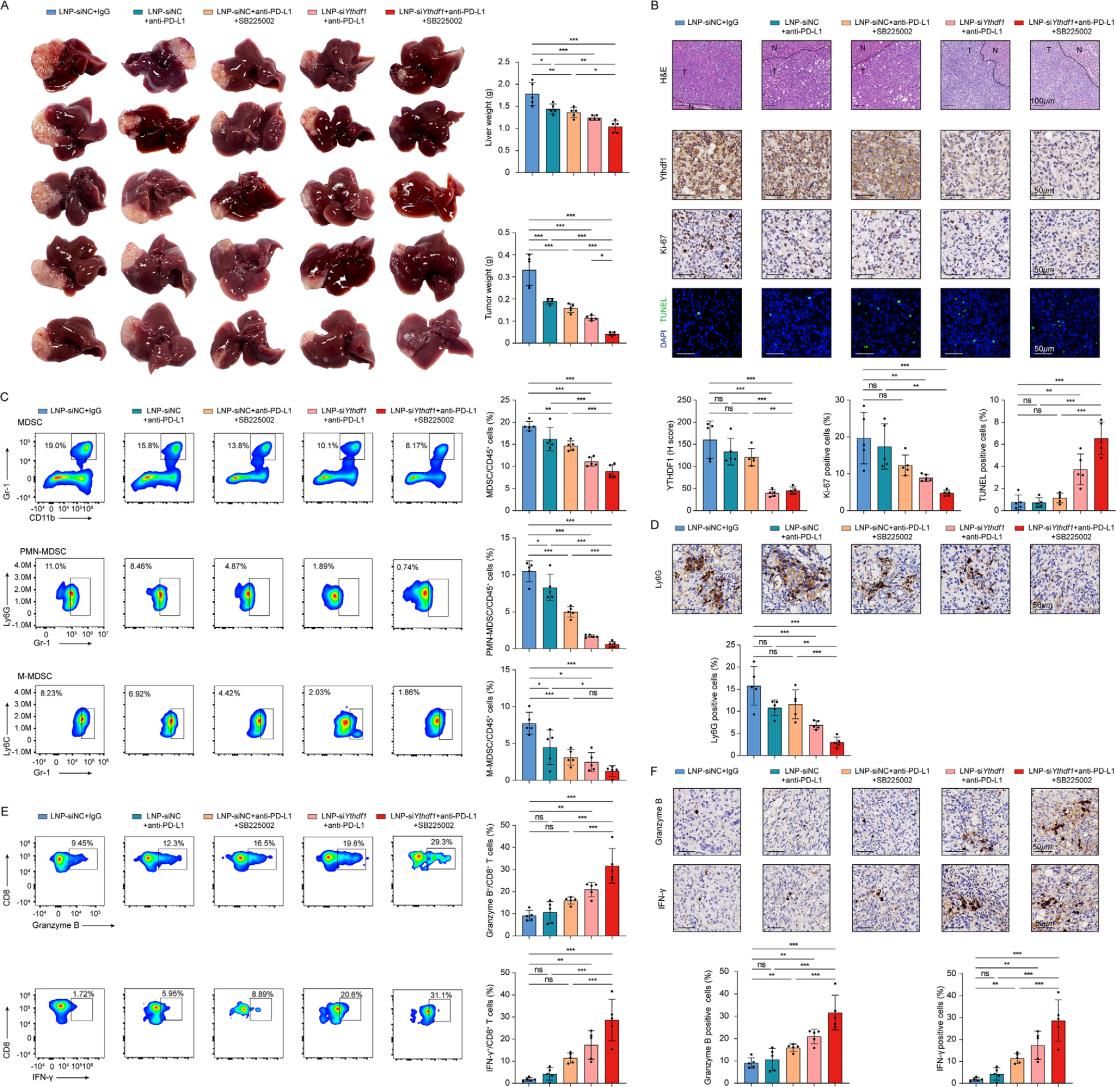
LNP‐si*Ythdf1* augments the efficacy of anti‐PD‐L1 therapy, which is further potentiated by CXCR2 inhibition. (A) Gross liver pathological photographs and statistical analysis of the orthotopic ICC model established by LTP‐C9 tumor cell orthotopic implantation and the combination therapy of LNP‐si*Ythdf1*, SB225002, and anti‐PD‐L1 (*n* = 5). (B) Representative images of H&E, IHC staining (YTHDF1, Ki67), TUNEL staining, and statistical analysis of ICC tumors in the orthotopic model with the indicated treatment (*n* = 5). (C) Flow cytometry analysis of MSDCs (upper), PMN‐MDSCs (median), and M‐MDSCs (lower) in orthotopic tumors with the indicated treatment (*n* = 5). (D) Representative IHC staining and quantification of Ly6G in orthotopic tumors with the indicated treatment (*n* = 5). (E) Flow cytometry analysis of Granzyme B^+^ (upper) and IFN‐γ^+^ (lower) CD8^+^ T cells in orthotopic tumors with the indicated treatment (*n* = 5). (F) Representative IHC staining and quantification of Granzyme B (upper) and IFN‐γ (lower) in orthotopic tumors with the indicated treatment (*n* = 5). Data are presented as means ± SD, by one‐way Anova (A–F); ns, no significance; *p < 0.05; **p < 0.01; ***p < 0.001.

To explore a more prospective therapeutic regime for precision medicine to eradicate ICC, we further explored whether depletion of YTHDF1 in combination with CXCR2 blockade, which inhibited PMN‐MDSC migration, could improve anti‐PD‐L1 efficacy. In the established orthotopic ICC mouse model, LNP‐si*Ythdf1*/siNC, SB225002, or vehicle control was administered in combination with PD‐L1 blockade or the control antibody. Then we performed tumor measurement and immune cell profiling at the time of sacrifice. As expected, triple blockade of YTHDF1, CXCR2, and PD‐L1 remarkably suppressed tumor growth, as evidenced by the liver gross images and tumor weight (Figure 6A). H&E and IHC staining confirmed the presence of tumor lesions and validated the *Ythdf1*‐knockdown efficiency (Figure 6B). Moreover, in the triple blockade group, we observed markedly reduced tumor cell proliferation and increased apoptosis in liver tissues (Figure 6B). Mechanistically, the greatest reduction in tumor‐infiltrating MDSCs, particularly the PMN‐MDSC subset, was achieved with triple blockade, surpassing the effect of LNP‐si*Ythdf1* plus anti‐PD‐L1 treatment (Figure 6C,D). Notably, the most pronounced enhancement of effector functions in tumor‐infiltrating CD8^+^ T cells was observed by the triple blockade therapy, as evidenced by flow cytometry and IHC staining (Figure 6E,F). Collectively, these findings demonstrate that concurrently targeting YTHDF1 and CXCR2 maximally potentiates anti‐PD‐L1 efficacy, highlighting a promising combinatorial strategy for ICC treatment.

## 3 Discussion

**FIGURE 7:**
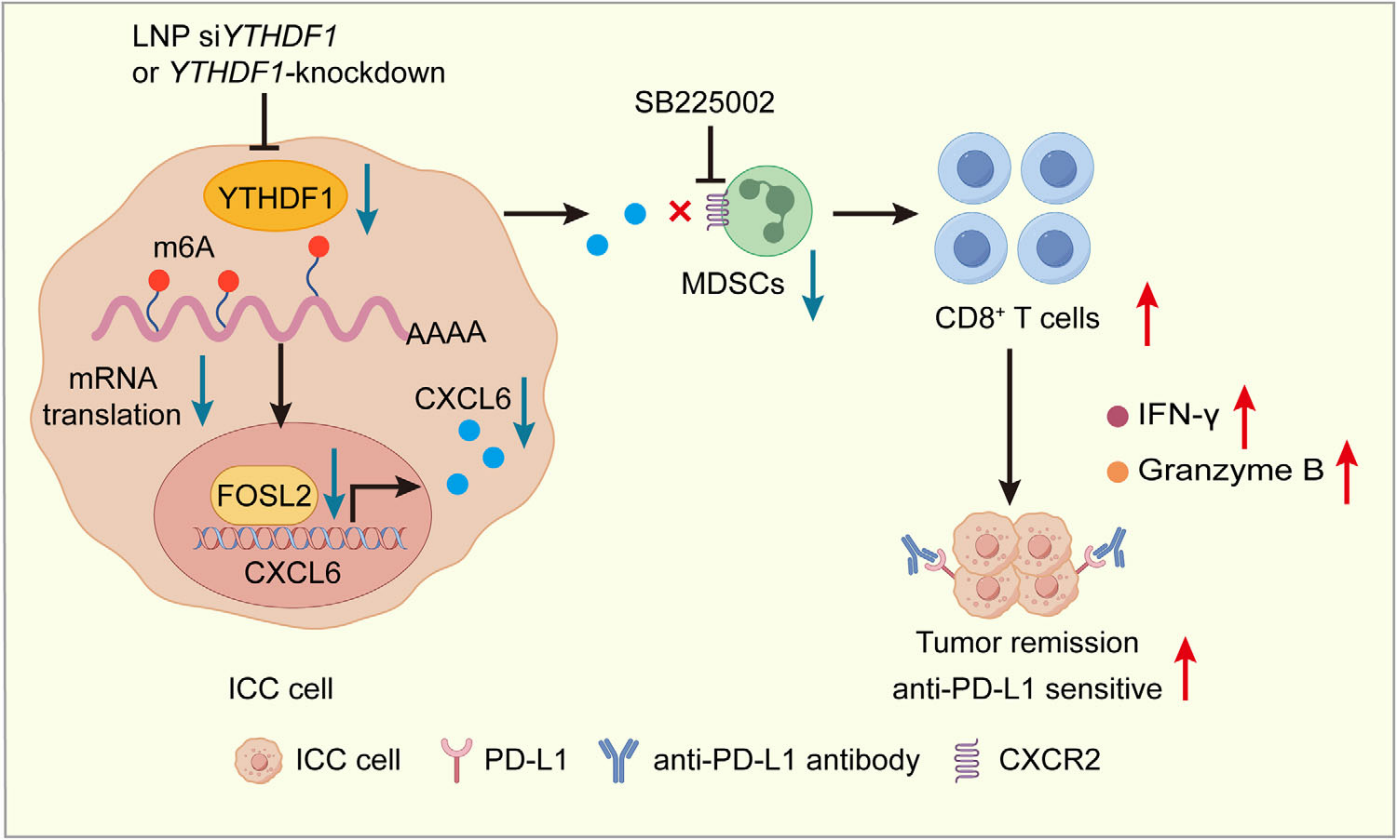
Schematic representations of YTHDF1 in ICC immunotherapy. YTHDF1 recruits PMN‐MDSCs to inhibit CD8^+^ T cells infiltration and function, and eventually promotes ICC progression. Mechanistically, YTHDF1 regulates MDSC accumulation via the FOSL2‐CXCL6‐CXCR2 axis. Targeting YTHDF1 improves the efficacy of anti‐PD‐L1 therapy of ICC.

ICC is an aggressive malignant disease with an alarming mortality rate which is often diagnosed at a late stage and lacks effective therapeutic options. Gemcitabine and cisplatin chemotherapy plus a PD‐L1 inhibitor has been recommended as the first‐line regimen of advanced ICC, but the response rate was very low [29, 30]. Thus, there is an urgent need to unravel molecular targets that sensitize ICC to immunotherapy. Here, we identified that YTHDF1 recruits and activates MDSCs, especially PMN‐MDSCs, thereby suppressing CD8^+^ T cell infiltration and function, eventually promoting ICC progression. Mechanistically, YTHDF1 induces chemokine CXCL6 secretion and promotes MDSC migration to ICC via binding to its receptor CXCR2. Targeting YTHDF1 and CXCR2 synergize with anti‐PD‐L1 therapy, obviously suppressed ICC tumor growth (Figure 7).

Expanding evidence underscores the pivotal role of the m^6^A epi‐transcriptome across multiple hallmarks of cancer, including modulation of the TIME [31, 32]. Within the m^6^A methyltransferase complex, the catalytic core component METTL3 has been shown to promote an immunosuppressive microenvironment in bladder cancer through m^6^A‐dependent regulation of CXCL5 and CCL5 [33]. In parallel, m^6^A demethylases also contribute to immune regulation; for instance, ALKBH5 facilitates tumor progression in head and neck squamous cell carcinoma by suppressing RIG‐I expression and interferon‐α production via the IKKε/TBK1/IRF3 pathway [34].

Despite these advances, the role of m^6^A reader proteins in shaping tumor immunity remains less clearly defined. Among them, YTHDF1 emerges as a dominant and uniquely dysregulated factor in ICC. While YTHDF1 has previously been linked to dendritic cell dysfunction in melanoma [35], our study provides the first evidence that YTHDF1 acts as a principal driver of the immunosuppressive microenvironment in ICC. Mechanistically, we demonstrated that YTHDF1 serves as a pivotal mediator that transduces m^6^A signals into transcriptional activation of the FOSL2‐CXCL6 axis, thereby promoting the establishment of an MDSC‐enriched, immunosuppressive microenvironment in ICC.

Tumor cells have been well‐documented to recruit MDSCs to shape an immunosuppressive microenvironment by production of cytokines and chemokines, leading to resistance to immunotherapy and negative prognosis [36, 37]. During chronic inflammation and cancer, myeloid cell precursors proliferated and differentiated into MDSCs and migrated into the tumor microenvironment. MDSCs were divided into two groups: PMN‐MDSCs and M‐MDSCs, which were mainly induced by G‐CSF and GM‐CSF, respectively [15, 38]. The immunosuppressive activity associated with MDSCs involves some factors, including ARG1, NO, ROS and prostaglandin E2 (PGE2) [39, 40, 41, 42], the changes in oxidative phosphorylation and glycolysis in tumors are also manifestations of MDSCs functions [43]. MDSCs would mature into tumor‐associated macrophages (TAMs), promote the formation of T regulatory cells (Tregs), which are critical for immune inhibition, and promote differentiation of fibroblasts to cancer‐associated fibroblasts (CAFs) [44, 45]. These terminally differentiated myeloid cells can persist in tissues for a long time and inhibited the CD8^+^ T cells immune response. In tumors, MDSCs suppress the proliferation and secretion of effector factors, such as IFN‐γ and Granzyme B of CD8^+^ T cells and induce their apoptosis [42]. Hence, understanding the molecular mechanisms that regulate the accumulation and function of these cells is necessary for precise cancer therapy.

Chemokines control the trafficking of immune cells and exert their biological function through binding to chemokine receptors [46, 47]. Here, we uncovered that YTHDF1 induces CXCL6 expression and secretion in ICC cells, promoting the migration of MDSCs, thereby inhibits the proliferation and functions of CD8^+^ T cells. The tumor progression was impaired by antagonizing the CXCL6 receptor CXCR2 in ICC tumor‐bearing mice. The effect of YTHDF1 on CXCL6 was also validated in human ICC tumors, which exhibited a positive correlation between YTHDF1 and CXCL6 protein expression. *Ythdf1*‐CKI mice also showed that Ythdf1‐overespression increased the Cxcl6 secretion in tumor tissues with ELISA.

We next elucidated the mechanistic link between YTHDF1 and CXCL6. Integrative multiomic sequencing revealed that FOSL2 is the primary target of YTHDF1 that promotes CXCL6 expression in ICC cells. FOSL2 is a member of the Fos transcription factor family, by heterodimerizing with Jun family members to form the AP‐1 transcription factor. By modulating the transcription of target genes, FOSL2 mediates a diversity of biological processes including growth, epithelial‐mesenchymal transition, invasion, metastasis formation, and drug resistance of cancer cells [48]. Moreover, FOSL2 has been observed to play a crucial role in TIME remodeling. In pancreatic ductal adenocarcinoma (PDAC) cells, FOSL2 promotes the transcription and secretion of chemokine CCL28, which recruits regulatory T cells and forming a suppressive immune microenvironment [49]. In colorectal cancer, FOSL2 increases CXCL10 secretion, eventually promotes CD8^+^ T cell infiltration, and enhances cytotoxic response against tumor cells [50]. Our study demonstrated that YTHDF1 recognized and bound to m^6^A‐modified *FOSL2* mRNA and promotes its translation in ICC cells. FOSL2 then binds to the promoter region of CXCL6 and promotes its transcription and secretion. The secreted CXCL6 subsequently facilitates MDSC migration into the ICC microenvironment by interacting with its receptor, CXCR2. Taken together, YTHDF1 induces the m^6^A‐FOSL2‐CXCL6/CXCR2 axis to drive impaired immune response in ICC.

In our previous study, YTHDF1 is found to be significantly associated with shorter recurrence‐free survival of ICC patients [13]. In this study, immunostaining of human ICC tissue microarray revealed that YTHDF1 drives immunosuppression, which was positively related with increased MDSC accumulation and decreased CD8^+^ T cells infiltration. The response rate of ICI therapy in ICC patients remains very low [5]. This can be partially attributed to the accumulation of immunosuppressive MDSCs, in conjunction with the poor tumor infiltration of cytotoxic CD8^+^ T cells. Hence, we proposed a combinatorial strategy of co‐targeting Ythdf1 and CXCR2 plus anti‐PD‐L1 treatment for ICC. Our results showed that Ythdf1 and CXCR2 inhibition in ICC tumors, synergized with anti‐PD‐L1 therapy inhibited tumor progression and infiltration of MDSCs, enhancing CD8^+^ T cell antitumor response. Our study suggested YTHDF1 as a potential therapeutic target for combination ICI therapy for ICC.

In conclusion, YTHDF1 drives an immunosuppressive microenvironment which recruits MDSCs and inhibits cytotoxic effector CD8^+^ T cells, thereby promoting ICC progression. Mechanistically, YTHDF1 promotes MDSC recruitment via activating the m^6^A‐FOSL2‐CXCL6/CXCR2 axis. Targeting YTHDF1 improves the antitumor efficacy of anti‐PD‐L1 therapy for ICC, highlighting YTHDF1 as a potential target for ICC immunotherapy.

## 4 Experimental Section

### 4.1 Patients’ Information

All ICC tissues were obtained from patients diagnosed with ICC at the First Affiliated Hospital, Sun Yat‐sen University (FAH‐SYSU). 291 ICC tissues were subjected to RNA‐sequencing (termed as SYSU cohort), and ICC tissue microarrays comprised of 179 ICC samples were used for immunohistochemistry staining. This study was approved by the Ethical Committee of the FAH‐SYSU (No.IIT‐2022‐131) and complied with all relevant ethical regulations. Written informed consents were provided by all patients.

### 4.2 Cell Lines and Cell Culture

The mouse cholangiocarcinoma cell line LTP‐C9 cells were established previously in our lab [51], which were derived from C57BL/6 mice after hydrodynamic injection with AKT, YAP, and Sleeping Beauty plasmids. The human HuCCT1 (RRID: CVCL_0324) and RBE (RRID: CVCL_4896) cell lines were obtained from Japan Health Sciences Foundation Resources Bank (Osaka, Japan) and Chinese Type Culture Collection (Shanghai, China), respectively. All the cells were cultured in DMEM (GIBCO) with 10% Fetal Bovine Serum (FBS) (GIBCO) and 1% Penicillin Streptomycin (GIBCO) at 37°C under a 5% CO_2_ atmosphere. All cell lines tested negative for mycoplasma contamination.

### 4.3 Plasmids Construction and Lentivirus Transduction

Short hairpin RNAs (shRNA) were synthesized with pLKO.1 vector from GENE, China. The shRNA sequences were as follows: sh*Ythdf1*‐1: 5’‐GGACATTGGTACTTGGGATAA‐3’; sh*Ythdf1*‐2: 5’‐GCACTGACTGGTGTCCTTT‐3’; sh*YTHDF1*‐1: 5’‐CCCGAAAGAGTTTGAGTGGAA‐3’; sh*YTHDF1*‐2: 5’‐CCCTACCTGTCCAGCTATTAC‐3’; sh*FOSL2*‐1: 5’‐CACGGCCCAGTGTGCAAGATT‐3’; sh*FOSL2*‐2: 5’–GCAGTGAGTATTGGAAGACTT‐3’; shFosl2‐1: 5’‐CCAGTCATCAGACTCCTTGAA‐3’; sh*Fosl2*‐2: 5’‐GCAGAAAGAGATTGCTGAGCT‐3’; sh*METTL3*‐1: 5’‐GCTGCACTTCAGACGAATTAT‐3’; sh*METTL3*‐2: 5’‐CGTCAGTATCTTGGGCAAGTT‐3’. The overexpression plasmids of WT YTHDF1, mutant YTHDF1 (K395A and Y397A), and FOSL2 were respectively cloned into the pCDH‐EF1‐MCS‐IRES‐Puro vector. To establish stable cell lines, HEK293T cells were seeded in a 10 cm dish and transfected with 12 µg of target plasmid, 8 µg of pCMV‐DR8.9, 4 µg of pCMV‐VSVG, and lipofectamine 3000 (L3000015, Life Technologies). Forty‐eight hours after transfection, the supernatant was collected and filtered by 0.45 µm membrane. The tumor cells were transfected with the lentivirus with 8 µg/mL polybrene (H8761, Solarbio), the stable transfected cells were selected with Puromycin (P8230, Solarbio).

### 4.4 AKT/YapS127A‐Induced Ythdf1‐Overexpressed ICC Mouse Model

Animal experiments were approved by the Institutional Animal Care and Use Committee of the FAH‐SYSU (No. [2022]015). Six‐week‐old C57BL/6 male mice were randomly divided into two groups, 6 mice per group. They were hydrodynamically injected with a mixture of 20 µg pT3‐EF1a‐HA‐myr‐AKT (Addgene, Cambridge, MA, USA), 30 µg pT3‐EF1a‐YapS127A (Addgene) along with 2.75 µg Sleeping Beauty transposase (pCMV/SB) plasmid (Addgene) via lateral tail vein [52, 53]. Additionally, 20 µg Ythdf1 plasmids were appended to the *Ythdf1‐*overexpressed group, and 20 µg vector plasmids were used in the control group. For interventional studies, mice were treated with 200 µg anti‐IgG (i.p. every fourth day, clone 2A3, BioxCell) or 200 µg anti‐Ly6G (i.p. every fourth day, clone 1A8, BioxCell) for PMN‐MDSC depletion, 2% DMSO (i.p. 6 days a week) or 2 mg/kg SB225002 (i.p. six days a week, S7651, Selleck) for CXCR2 inhibition. Mice were sacrificed four weeks after injection, and liver tissues were harvested for flow cytometry and IHC staining.

### 4.5 Liver‐Specific Ythdf1‐Knockin Transgenic Mice

The liver‐specific *Ythdf1* overexpressing transgenic /*Alb*‐Cre mice were described before [11]. Briefly, loxp‐stop‐loxp (*lsl)*‐*Ythdf1* mice were crossed with *Alb*‐cre mice to generate *Ythdf1* transgenic /*Alb*‐Cre mice (hereinafter termed as *Ythdf1* CKI mice). Littermate *lsl*‐*Ythdf1* mice were used as control (hereafter termed as Ctrl mice). All the mice were confirmed by PCR genotyping. The *Ythdf1* CKI and Ctrl mice were subjected to establish ICC models by hydrodynamical injection with 20 µg myr‐AKT, 30 µg YAP, and 2.75 µg pCMV‐SB plasmids. The mice were sacrificed at four weeks post‐injection or humane endpoint.

### 4.6 Orthotopic ICC Mouse Model

For the liver‐orthotopic ICC mouse model, six‐week‐old male C57BL/6 mice were randomized into two groups, nine mice in each group. LTP‐C9 cells (3 × 10^6^; shNC or sh*Ythdf1*) in 20 µL PBS were injected into the left liver lobe. For interventional studies, mice were randomized and treated with 200 µg anti‐IgG (i.p. every fourth day for four weeks, clone 2A3, BioxCell) or 200 µg anti‐PD‐L1 (i.p. every fourth day for four weeks, clone 10F.9G2, BioxCell) for PD‐L1 blockade after tumor cell implantation for two weeks. Mice were sacrificed six weeks after tumor inoculation, and the tumors were collected for further analysis.

### 4.7 siRNA Treatment Packaged by Lipid Nanoparticle (LNP)

Small interfering RNA (siRNA) targeting Ythdf1 with 2’ O‐Methyl (2’‐OMe) modification was synthesized from Tsingke Biotechnology Company (Beijing, China). The sequence of si*Ythdf1* we used was as follows: 5’‐GCACTGACTGGTGTCCTTT‐3’. siRNAs were packaged into LNP by Guangzhou Kelan Biotechnology (Guangzhou, China). LNP‐siRNA was used to treat the mice two weeks after tumor inoculation at a dose of 1 mg/kg every three days for twelve days. Mice were sacrificed six weeks after tumor inoculation.

### 4.8 Triple Blockade of YTHDF1, CXCR2 and Anti‐PD‐L1 Mice

Treatments were initiated two weeks after orthotopic tumor implantation. LNP‐si*Ythdf1* or LNP‐siNC was administered as previously described. Mice additionally received either 2% DMSO (i.p., 6 days per week) or the CXCR2 inhibitor SB225002 (2 mg/kg, i.p., 6 days per week; S7651, Selleck). For immune checkpoint blockade, mice were treated with anti‐PD‐L1 (200 µg per mouse, i.p., every 4 days for 4 weeks; clone 10F.9G2, Bio X Cell) or isotype control IgG (200 µg per mouse, i.p., every 4 days for 4 weeks; clone 2A3, Bio X Cell). Mice were sacrificed six weeks after tumor inoculation.

### 4.9 Western Blotting

Cells or tissues were lysed with RIPA lysis buffer (PC101, EpiZyme) supplemented with protease inhibitor (#4693159001, Roche) and phosphatase inhibitor (#4906837001, Roche). Twenty micrograms of proteins were separated with polyacrylamide gel electrophoresis (PAGE) and transferred onto a 0.45 µm polyvinylidene difluoride (PVDF) membrane. After blocking with 5% skim milk, the membranes were incubated with primary antibodies at 4°C overnight. Primary antibodies used in this study were as follows: anti‐GAPDH (2118S, CST), anti‐YTHDF1 (17479‐1‐AP, Proteintech), anti‐FOSL2 (sc‐166102, Santa Cruz Biotechnology), and anti‐METTL3 (ab195352, Abcam). Next, the membrane was incubated with HRP‐linked secondary antibodies and visualized by a multifunction imaging system.

### 4.10 Hematoxylin‐Eosin (H&E), Immunohistochemistry (IHC), Immunofluorescence (IF) Staining

H&E, IHC, and IF staining were performed with 4 µm paraffin‐embedded tissue sections. All the slides were dewaxed and hydrated. For H&E staining, the sections were stained with hematoxylin and eosin for 3 min (E607318, Sangon Biotech). For IHC staining, sections were subjected to heat‐induced epitope retrieval and blocked with 20% goat serum for 30 min. The primary antibodies were incubated with tissues overnight at 4°C. The primary antibodies we used were as follows: anti‐YTHDF1 (17479‐1‐AP, Proteintech), anti‐Ki‐67 (12202, CST), anti‐CD3 (Ab16669, Abcam), anti‐CD8 for mouse (ab209775, Abcam), anti‐CD8 for human (70306S, CST), anti‐CK19 (ab169526, Abcam), anti‐Ly6G (87048S, CST), anti‐Granzyme B (ab4059, Abcam), anti‐Interferon‐γ (ab9657, Abcam), anti‐FOSL2 for human (19967, CST), and anti‐FOSL2 for mouse (sc‐166102, santa cruz biotechnology). The HRP‐conjugated secondary antibody was incubated for 30 min the following day. After hematoxylin staining, sections were visualized with DAB Kit (K5007, Dako). For IF staining, the primary antibodies of anti‐CD15 (ab665, Abcam) and CD11b (ab133357, Abcam) were used overnight at 4°C followed by secondary antibody incubation for 1 h. Finally, DAPI was stained for 5 min, and the images were captured by OlyVIA.

### 4.11 TUNEL Staining

For TUNEL fluorescence staining, the In Situ Cell Death Detection kit (Biosharp, BL645C) was used following the manufacturer's standard protocol. Slices were incubated in 50 µL TUNEL reaction mixture (45 µL buffer, 1 µL Biotin‐dUTP mixed with 4 µL TdT Enzyme Solution) at 37°C in a dark wet box for 1 h, followed by DAPI stained for 5 min, and the images were captured by OlyVIA.

### 4.12 Chemokine Array

Human HuCCT1 sh*YTHDF1*‐1, sh*YTHDF1*‐2, and their corresponding control cells were seeded at equal densities and cultured in DMEM supplemented with 10% FBS for 48 h. Conditioned media were collected and centrifuged twice at 3000 × g for 10 min at 4°C to remove cellular debris. The supernatants were harvested and subjected to cytokine multiplex immunoassays following appropriate and equal dilution. Chemokine profiling was performed using the Bio‐Plex Pro Human Chemokine Panel (Bio‐Rad) for YTHDF1 knockdown experiments, according to the manufacturers’ instructions.

### 4.13 Enzyme‐Linked Immunosorbent Assay (ELISA)

Conditioned medium was collected from the indicated tumor cells culture in DMEM with 10% FBS for 72 h. Culture supernatants were collected with a centrifuge with 3000 g, 10 min at 4°C. The production of CXCL6 was analyzed by Mouse LIX Quantikine ELISA Kit (MX000, R&D Systems) or Human CXCL6/GCP‐2 ELISA Kit (BSEH‐214, biosharp). The production of CXCL10, CXCL11, and CCL20 was respectively analyzed by Human CXCL10/IP10 ELISA KIT (BSEH‐091, biosharp), Human CXCL11/I‐TAC ELISA KIT (BSEH‐213, biosharp), and Human MIP‐3α/CCL20 ELISA KIT (BSEH‐061, biosharp) according to the manufacturers’ protocol.

### 4.14 MDSC Isolation and Migration Assay

Mouse MDSCs were isolated from the spleens of LTP‐C9 ICC orthotopic tumor‐bearing mice using EasySep Mouse MDSC (CD11b^+^Gr1^+^) Isolation Kit (19867, STEMCELL). Conditioned medium was collected form LTP‐C9 shNC or sh*Ythdf1* cells being cultured for 48 h in 10% FBS. MDSCs (1 × 10^5^) were seeded into the top chamber of the Transwell (pore size: 3 µm, 3415, Corning), the bottom chamber was filled with the indicated conditioned medium. Cells were incubated for 4 h at 37°C under a 5% CO_2_ atmosphere, the migrated MDSCs in the bottom were counted.

### 4.15 Flow Cytometry

Primary cells were isolated from fresh tumor tissues by mechanical dissection through 70 µm nylon cell strainers, cells were centrifuged at 500 g for 5 min, and washed by PBS with 1% heat induced FBS. Red blood cells (RBCs) were removed by the RBC lysis buffer. Next, cells were incubated with L/D marker for 15 min at room temperature, followed by incubation with Fc Receptor Blocking Solution (553 141, BD Biosciences) for 15 min at 4°C. Surface staining was performed for 30 min at 4°C before fixation. For intracellular staining, CD8^+^ T cells were fixed and permeabilized using BD Fixation and Permeabilization Solution (554 722, BD). CD8^+^ T cells were stimulated by Leukocyte Activation Cocktail with BD GolgiPlugTM (550583, BD Biosciences) for 4–6 h at 37°C before incubation with L/D marker. Intracellular staining was performed at 4°C for one hour. The antibodies used for flow cytometry were as follows: Fixable Viability Stain 700 (564 997, BD Biosciences), anti‐CD45‐BUV395 (564 279, BD Biosciences, 1:400), anti‐CD11b‐FITC (Biolegend, 101 206, 1:100), anti‐Gr1‐BV786 (740 850, BD Biosciences, 1:200), anti‐Ly6C‐APC (128 016, Biolegend, 1:200), anti‐Ly6G‐PE/Cy7 (560601, BD Biosciences, 1:200), anti‐CD3e‐APC‐Cy7 (557596, BD Biosciences, 1:200), anti‐CD8‐Percp‐Cy5.5 (100734, Biolegend, 1:200), anti‐IFN Gama‐BV480 (564336, BD Biosciences, 1:100), anti‐Granzyme B‐Pacific blue (25‐8898‐82, Thermo Fisher scientific, 1:100). Flow cytometry was performed with Cytek Aurora (Cyted Biosciences, CA) and data were analyzed with FlowJo V10 software.

### 4.16 T Cell Proliferation Assay

CD8^+^ T cells were isolated from the spleens of nontumor‐bearing C57BL/6 mice using EasySep Mouse CD8^+^ T‐cell Isolation Kit (19 853, STEMCELL) according to the manufacturer's instructions. For the T Cell Suppression Assay which MDSCs were co‐cultured with CD8^+^ T cells, MDSCs were separated from shNC and sh*Ythdf1* allografts using EasySep Mouse MDSC (CD11b^+^Gr1^+^) Isolation Kit (19867, STEMCELL). The isolated MDSCs were co‐cultured with 1 µm carboxyfluorescein diacetate succinimidyl ester (CFSE)‐labeled CD8^+^ T cells at a ratio of 1:1 in RPMI 1640 medium containing DynabeadsTM Mouse T‐Activator CD3/CD28 (11456D, Thermo Fisher Scientific) and recombinant IL‐2 (200‐02, Peprotech) for 72 h. CFSE intensity was quantified by flow cytometry to assess T cell proliferation. To evaluate the function of CD8^+^ T cells, CD8^+^ T cells were collected and stained with anti‐CD3e‐APCCy7, anti‐CD8‐Percp‐Cy5.5, anti‐IFN Gama‐BV480, and anti‐Granzyme B‐Pacific blue antibodies and analyzed by flow cytometry. For the CD8^+^ T cell group, CFSE‐labeled CD8^+^ T cells were cultured with tumor‐conditioned medium containing DynabeadsTM Mouse T‐Activator CD3/CD28 (11456D, Thermo Fisher Scientific) and recombinant IL‐2 (200‐02, Peprotech) for 72 h, then the cells were subjected to flow cytometry analysis.

### 4.17 Reverse Transcription‐Quantitative Polymerase Chain Reaction (RT‐qPCR)

Total RNA was extracted by TRIzol reagent (Invitrogen), and 1 µg RNA was used to synthesize cDNA using PrimeScript RT reagent kit (RR037Q, Takara) according to the manufacturer's instructions. RT‐qPCR analysis was performed with TB Green Premix Ex Taq II (RR820A, Takara) on Applied Biosystems QuantStudio 5. GAPDH was used as an internal control. Relative expression levels were calculated using the 2^−^ *^ΔΔCt^* method.

### 4.18 RNA Sequencing (RNA‐Seq)

For human samples, a total of 291 tumor samples from ICC patients collected in our center were subjected to RNA‐seq analysis, from which 66 cases were previously reported by Chen Shuling et al. [54], 157 cases were documented in our previous research [55], and the remaining samples are newly included in this study. All patients provided written informed consents. For ICC cell lines, YTHDF1‐knockdown, YTHDF1‐overexpressing, and corresponding control HuCCT1 cells were used for RNA‐seq. For RNA‐seq, total RNA was extracted using Trizol reagent, and the enriched mRNA was fragmented into short fragments and reverse transcribed into cDNA by using NEBNext Ultra RNA Library Prep Kit for Illumina (7530, New England Biolabs, USA). The RNA‐seq libraries were sequenced on the Illumina NextSeq 500 platform (Illumina). The FPKM (fragment per kilobase of transcript per million mapped reads) value analyzed by DESeq2 was used to calculate RNA differential expression. Pathway enrichment analysis was conducted by the KEGG database and the GSEA database.

### 4.19 RNA Immunoprecipitation‐Sequencing (RIP‐Seq) and RIP‐qPCR

RNA‐binding protein immunoprecipitation assay was performed using Magna RIP RNA‐Binding Protein Immunoprecipitation kit (17–700, Merck) according to the manufacturer's instructions. Briefly, 2 × 10^7^ HuCCT1 cells were lysed with RIP lysis buffer on ice for 5 min, and 10 µL supernatant was kept as input. Magnetic beads were pre‐incubated with 5 µg anti‐YTHDF1 antibody (17479‐1‐AP, Proteintech) or normal Rabbit anti‐IgG antibody, then incubated with the RIP lysate supernatants with rotation overnight at 4°C. The binding RNA was purified and subjected to RIP‐qPCR or RIP‐seq.

### 4.20 Chromatin Immunoprecipitation (ChIP)

Chromatin immunoprecipitation was performed with Chromatin immunoprecipitation (ChIP) Kit (Bes5001, BersinBio) according to the manufacturer's instructions. In brief, 2 × 10^7^ cells were fixed by 1% formaldehyde, washed, and collected by centrifugation. After crosslinking, the precipitation was washed and applied to the lysis buffer to extract the nuclear contents. The DNAs were further sonicated to obtain appropriate fragments. The samples were centrifuged, and shared chromatin was used as input and incubated with Fra2 Antibody (G‐5) X (sc‐166102 X, Santa Cruz Biotechnology). Rabbit IgG was used as an isotype control. The binding complexes were washed, inverse‐crosslinked, eluted, and then evaluated by RT‐qPCR.

### 4.21 Ribosome Nascent‐Chain Complex‐Bound mRNA‐qPCR (RNC‐qPCR)

Cells (5 × 10^6^) were incubated with cycloheximide at a concentration of 100 µg/mL for 15 min at 37^°^C. Lysis buffer was prepared by adding 1% Triton X‐100 into ribosome buffer (RB buffer): 20 mm HEPES (pH = 7.4), 15 mm MgCl_2_, 200 mm KCl, 100 mg/mL cycloheximide, and 2 mm dithiothreitol). Cell lysates were extracted by centrifuging at 4^°^C for 10 min at 16 200 g. Two hundred microliters of supernatant were collected to extract total RNA. The other 1 mL supernatants were removed onto 35 mL sucrose buffer and then centrifuged at 174 900 g for 5 h at 4^°^C in a SW32 rotor to obtain RNC RNA. Total RNA isolated from the input control and RNC samples were used for RT‐qPCR for further analysis.

### 4.22 Polysome Profiling

Cells (1 × 10^7^) pretreated with 100 µg/mL cycloheximide at 37^°^C for 15 min were collected quickly using a cell scraper and extracted with 1 mL polysome lysis buffer at 4^°^C for 10 min. Then, suspensions were centrifuged at 13 000 g for 10 min and 1 mL supernatants were removed to the up‐layer of 10%–50% sucrose gradient. After centrifuged at 36 000 rpm for 2.5 h at 4^°^C. The fractions were lysed in TRIzol (Life Technologies), and the isolated RNA was used for further analysis by RT‐qPCR.

### 4.23 Statistical Analysis

Statistical analyses were conducted using GraphPad Prism 9 software. All experiments were performed at least in triplicate. Student's *t*‐test was employed to compare the differences between two groups, multiple comparisons were performed by one‐way ANOVA. The Kaplan–Meier survival analysis was performed for prognosis analysis. Data were presented as mean ± standard deviation (SD), The *p‐*values were represented as follows: *p < 0.05; **p < 0.01; ***p < 0.001.

## Author Contributions

L.L. and Z.L. performed the experiments and analyzed the data. L.L., Z.L., L.W., and W.C. drafted the manuscript. D.Z. performed the RNA‐sequencing analysis. C.L. contributed to the experiments of transgenic mouse models. F.F. helped with the in vitro experiments. K.L. assisted in the animal experiments. Z.S., S.S., and M.K. provided human samples and histological data. L.X., S.W., Y.J., and X.L. supervised the project and revised the paper. All authors reviewed and approved the final manuscript.

## Conflicts of Interest

The authors declare no conflicts of interest.

## Supporting information




**Supporting File**: advs74990‐sup‐0001‐SuppMat.docx

## Data Availability

The data that support the findings of this study are available from the corresponding author upon reasonable request.
