# Supplementary material for: Targeting m6A Reader YTHDF1 Enhances Antitumor Immunity and Potentiates Anti‐PD‐L1 Efficacy in Intrahepatic Cholangiocarcinoma
Source: Adv Sci (Weinh). 2026 Apr 13;13(32):e20403. doi: 10.1002/advs.202520403 (PMC13252639; doi:10.1002/advs.202520403)
Supplement: Supplementary file 1 — Supporting File: advs74990‐sup‐0001‐SuppMat.docx [file ADVS-13-e20403-s001.docx]

**Supporting Figures and Legends**

**
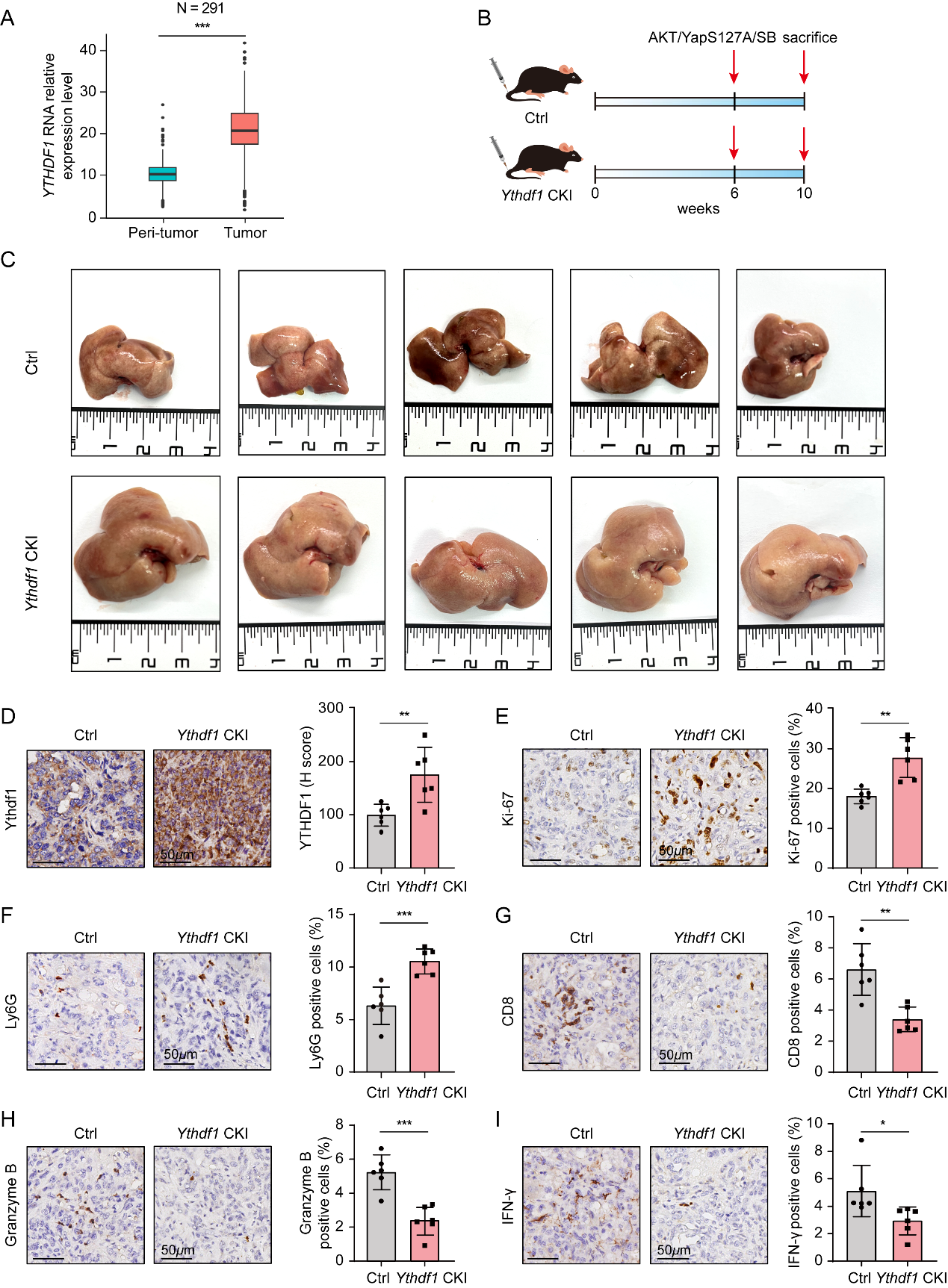
**

**Figure S1.** Hepatocyte-specific overexpression of Ythdf1 impairs ICC antitumor immunity. A) mRNA expression level of YTHDF1 in human ICC tumors versus peri-tumor tissues analyzed by RNA sequencing (SYSU cohort, n = 291). B) Schematic diagram of ICC induction via hydrodynamic tail vein injection of oncogenic plasmids in Ctrl and *Ythdf1* CKI mice. C) Gross liver pathological photographs of Ctrl and *Ythdf1* CKI mice. Remaining tumor images from indicated orthotopic tumor, supplementing the representative images shown in Figure 1C. D-I) Representative IHC staining (left) and quantification (right) of Ythdf1 (D), Ki-67 (E), Ly6G (F), CD8 (G), Granzyme B (H) and IFN-γ (I) in Ctrl and *Ythdf1* CKI ICC tumors (n = 6). Data are presented as means ± SD, by Student’s t test (A, D-I); **p* < 0.05; ***p* < 0.01; ****p* < 0.001.


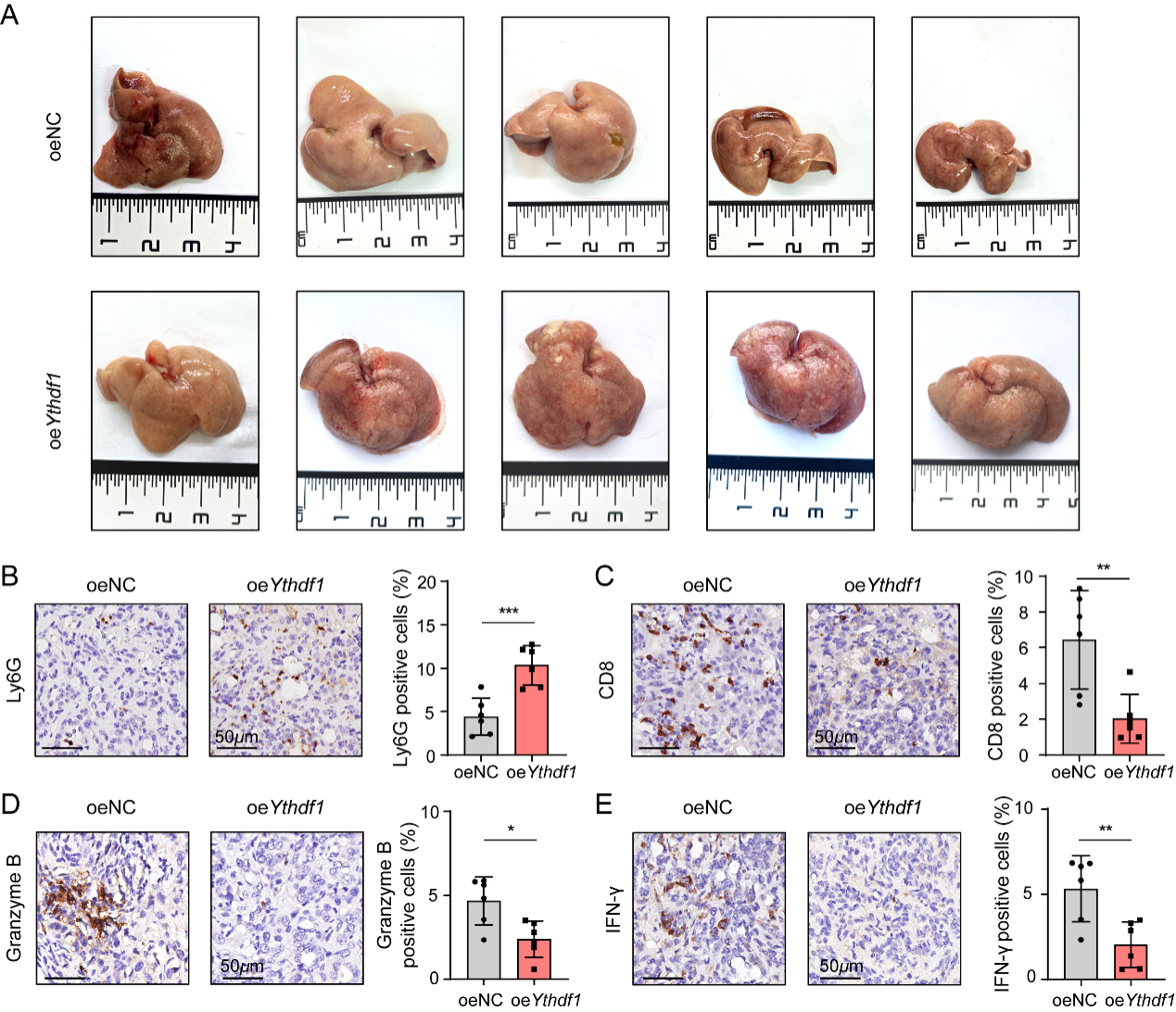


**Figure S2.** Ythdf1 overexpression affects the infiltration of PMN-MDSCs and CD8^+^ T cells. A) Gross liver pathological photographs of oeNC and oe*Ythdf1* mice. Remaining tumor images from indicated orthotopic tumor, supplementing the representative images shown in Figure 2B. B-E) Representative IHC staining (left) and statistical analysis (right) of Ly6G (B), CD8 (C), Granzyme B (D) and IFN-γ (E) expression in oeNC and oe*Ythdf1* hydrodynamic ICC mice model (n = 6). Data are presented as means ± SD, and by Student’s t test (B-E); **p* < 0.05; ***p* < 0.01; ****p* < 0.001.


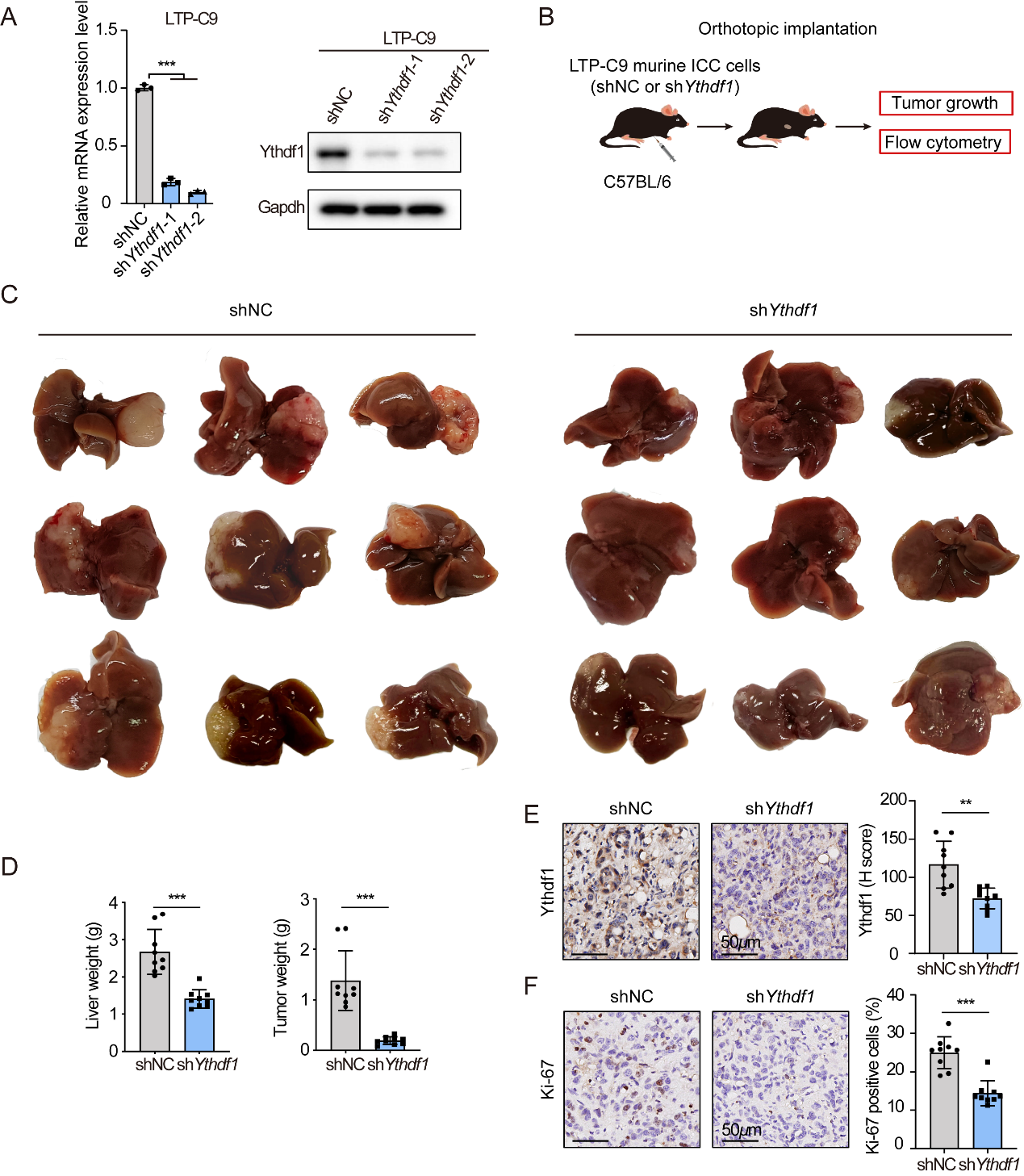


**Figure S3.** *Ythdf1*-knockdown suppresses orthotopic ICC tumor growth. A) RT-qPCR and Western blot validation of Ythdf1 knockdown in LTP-C9 cells. B) Schematic diagram of ICC orthotopic allograft mouse model using *Ythdf1*-knockdown or control LTP-C9 cells. C,D) Gross liver pathological photographs (C) and statistical analysis of liver weight as well as tumor weight (D) of shNC and sh*Ythdf1* mice. E,F) Representative IHC staining (left) and quantification (right) of Ythdf1 (E) and Ki-67 (F) expression in shNC and sh*Ythdf1* tumors (n = 9). Data are presented as means ± SD, by one-way anova (A), and by Student’s t test (D-F); **p* < 0.05; ***p* < 0.01; ****p* < 0.001.


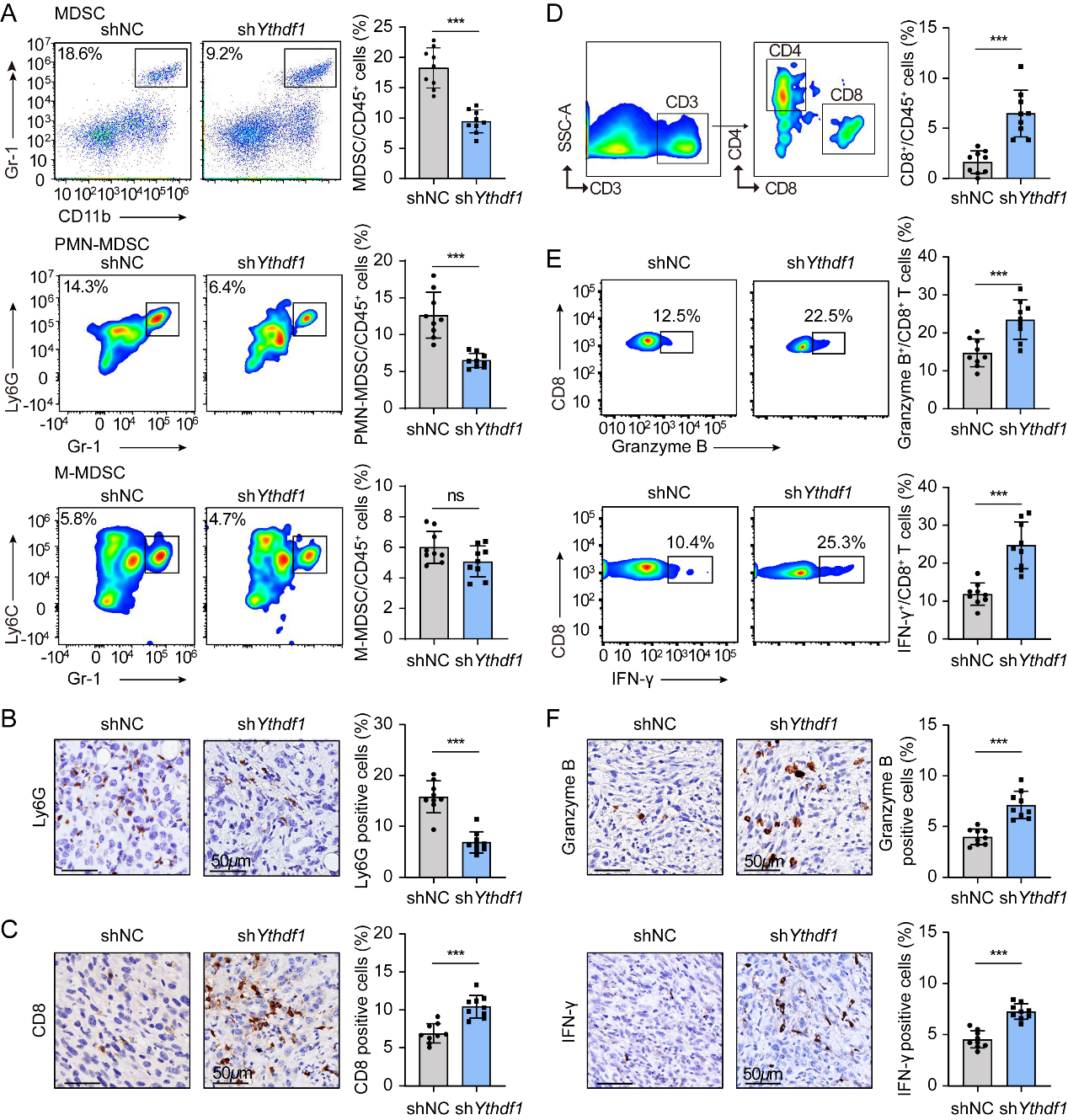


**Figure S4.** *Ythdf1*-knockdown induces antitumor immunity by reduction of MDSCs and increase of functional CD8^+^ T cells in ICC orthotopic tumors. A) Flow cytometric analysis of total MDSCs (upper), PMN-MDSCs (median) and M-MDSCs (lower) infiltration in shNC and sh*Ythdf1* tumors (n = 9). B,C) Representative IHC staining (left) and quantification (right) of Ly6G (B) and CD8 (C) expression in shNC and sh*Ythdf1* tumors (n = 9). D) Flow cytometry analysis of CD8^+^ T cells in shNC and sh*Ythdf1* tumors (n = 9). E) Flow cytometry analysis of Granzyme B^+^ (upper) and IFN-γ^+^ CD8^+^ (lower) T cells within CD8^+^ T cells in shNC and sh*Ythdf1* tumors (n = 9). F) Representative IHC staining (left) and quantification (right) of Granzyme B (upper) and IFN-γ (lower) expression in shNC and sh*Ythdf1* tumors (n = 9). Data are presented as means ± SD, by Student’s t test (A-F); **p* < 0.05; ***p* < 0.01; ****p* < 0.001.


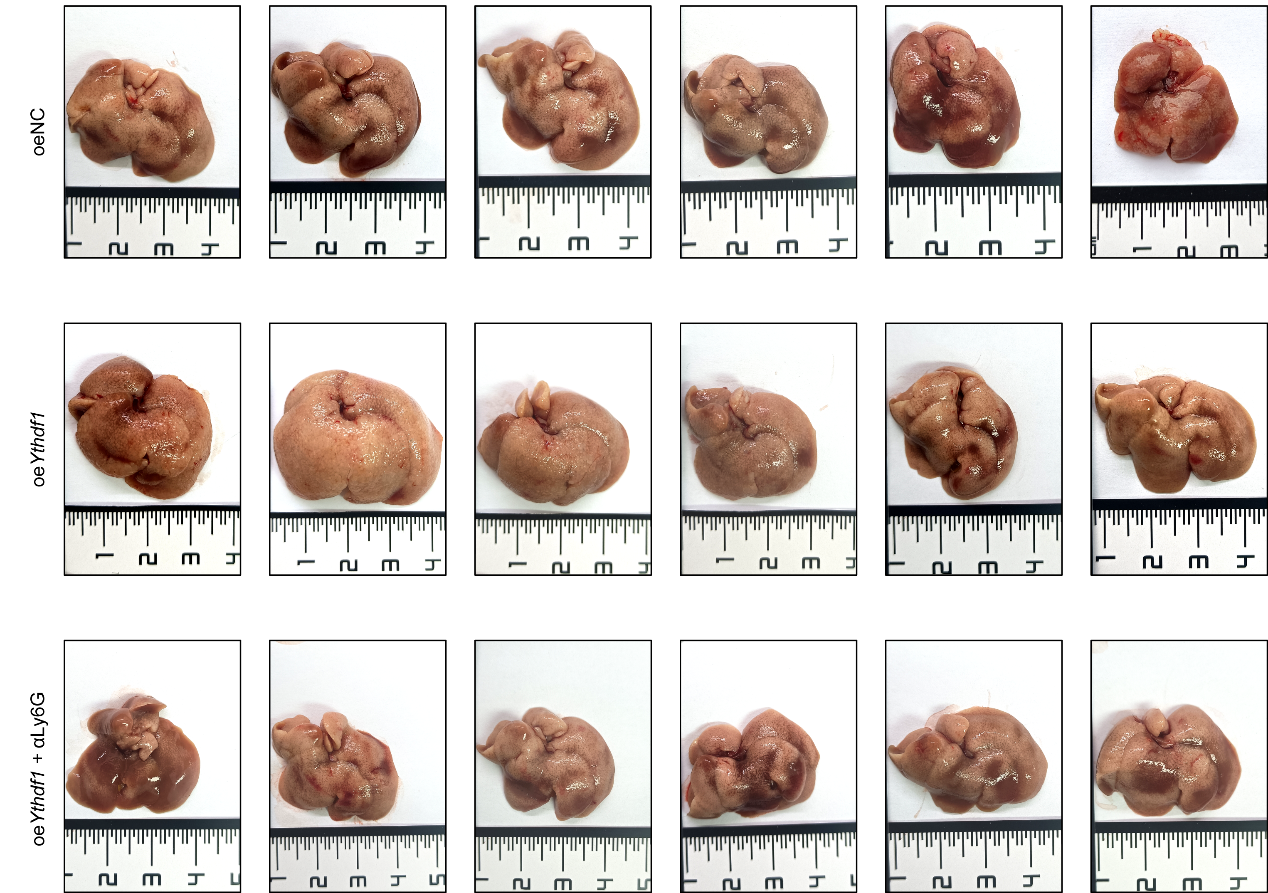


**Figure S5.** PMN-MDSC blockade greatly abolished the enhanced tumor growth by YTHDF1 overexpression. Gross liver pathological photographs of oeNC, oe*Ythdf1* and oe*Ythdf1* + αLy6G mice. Remaining tumor images from indicated orthotopic tumor, supplementing the representative images shown in Figure 3B.


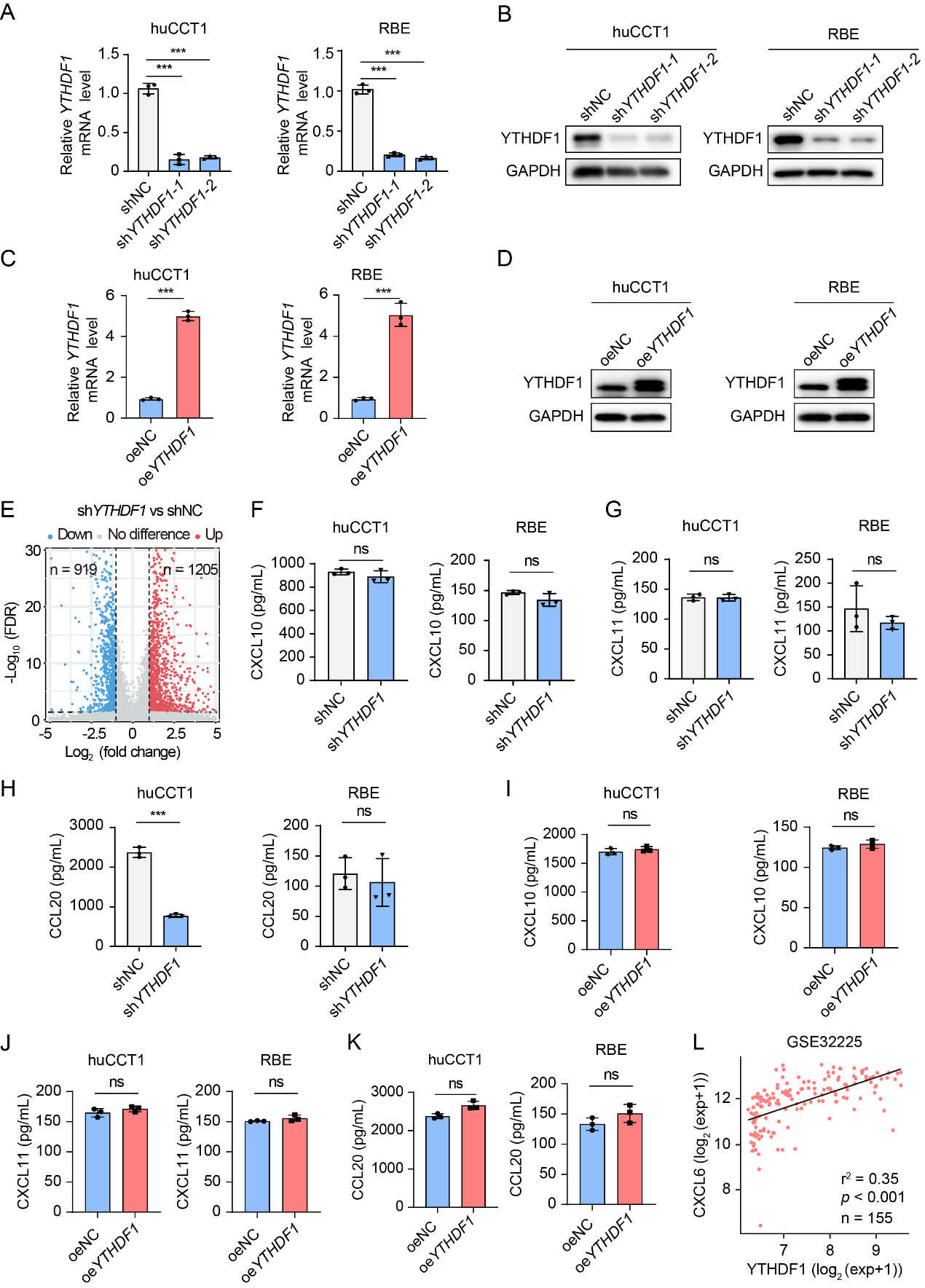


**Figure S6.** Overexpression of YTHDF1 upregulated CXCL6 expression in ICC. A,B) RT-qPCR (A) and Western blot (B) validated Ythdf1 knockdown efficiency in huCCT1 and RBE cells. C,D) RT-qPCR (C) and Western blot (D) validated Ythdf1 overexpression efficiency in huCCT1 and RBE cells. E) Differential gene expression analysis of *YTHDF1*-knockdown versus control huCCT1 cells by RNA-seq. F-H) ELISA analysis of CXCL10 (F), CXCL11 (G), and CCL20 (H) secretion in the conditioned medium of *YTHDF1*-knockdown and control huCCT1 and RBE cells (n = 3). I-K) ELISA analysis of CXCL10 (I), CXCL11 (J), and CCL20 (K) secretion in the conditioned medium of YTHDF1 overexpression and control huCCT1 and RBE cells (n = 3). L) Correlation analysis between YTHDF1 and CXCL6 expression in human cholangiocarcinoma samples from the GSE32225 dataset (n=155). Data are presented as means ± SD, by one-way anova (A), by Student’s t test (C, F-K), and by Spearman correlation test (L); ns, no significance; **p* < 0.05; ***p* < 0.01; ****p* < 0.001.


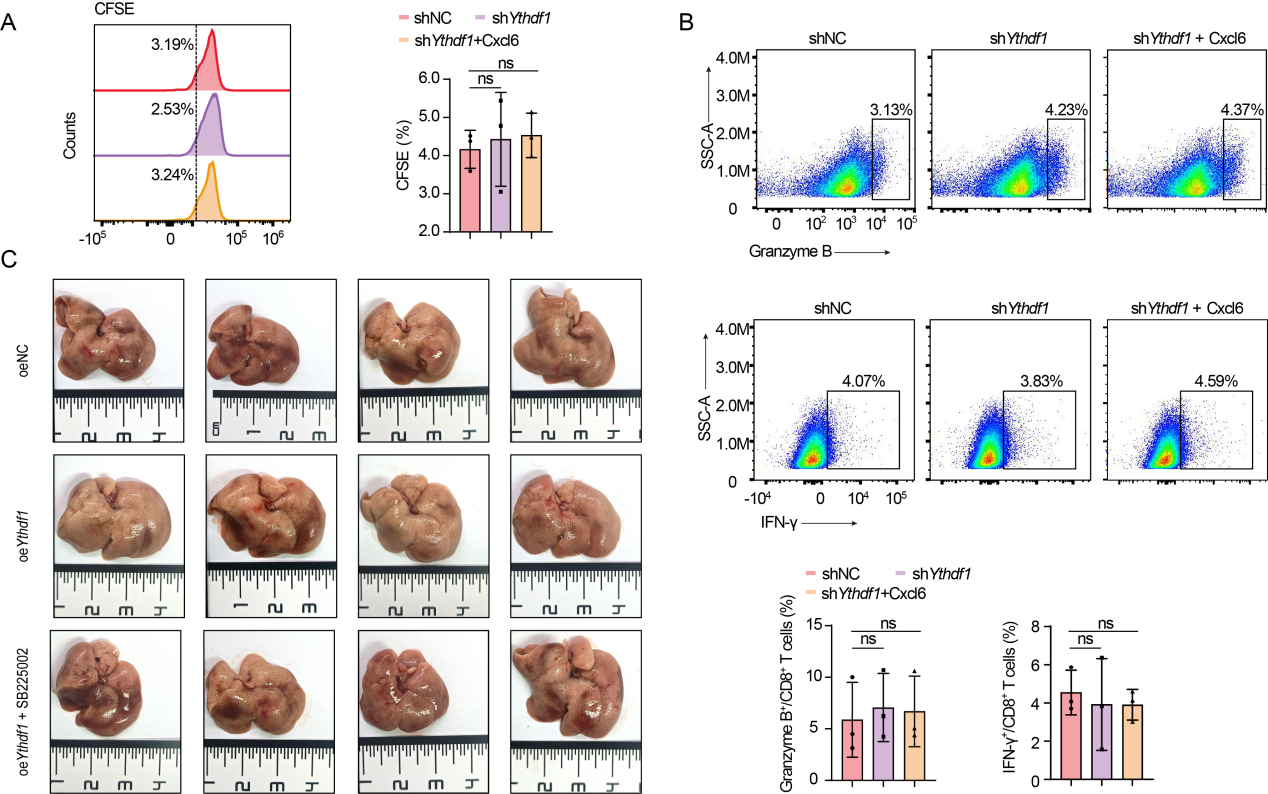


**Figure S7.** YTHDF1 recruits MDSCs via CXCL6 in ICC. A) CFSE-labeled CD8^+^ T cells were cultured with the indicated conditioned medium, and the proliferation rate of CD8^+^ T cells was determined by flow cytometry (n = 3). B) CD8^+^ T cells were cultured with the indicated conditioned medium, and the percentage of Granzyme B^+^ (upper) and IFN-γ^+^ (bottom) CD8^+^ T cells were analyzed by flow cytometry (n = 3). C) Gross liver pathological photographs of oeNC, oe*Ythdf1* and oe*Ythdf1* + SB225002 mice. Remaining tumor images from indicated orthotopic tumor, supplementing the representative images shown in Figure 3M. Data are presented as means ± SD, by one-way anova (A-B); ns, no significance; **p* < 0.05; ***p* < 0.01; ****p* < 0.001.


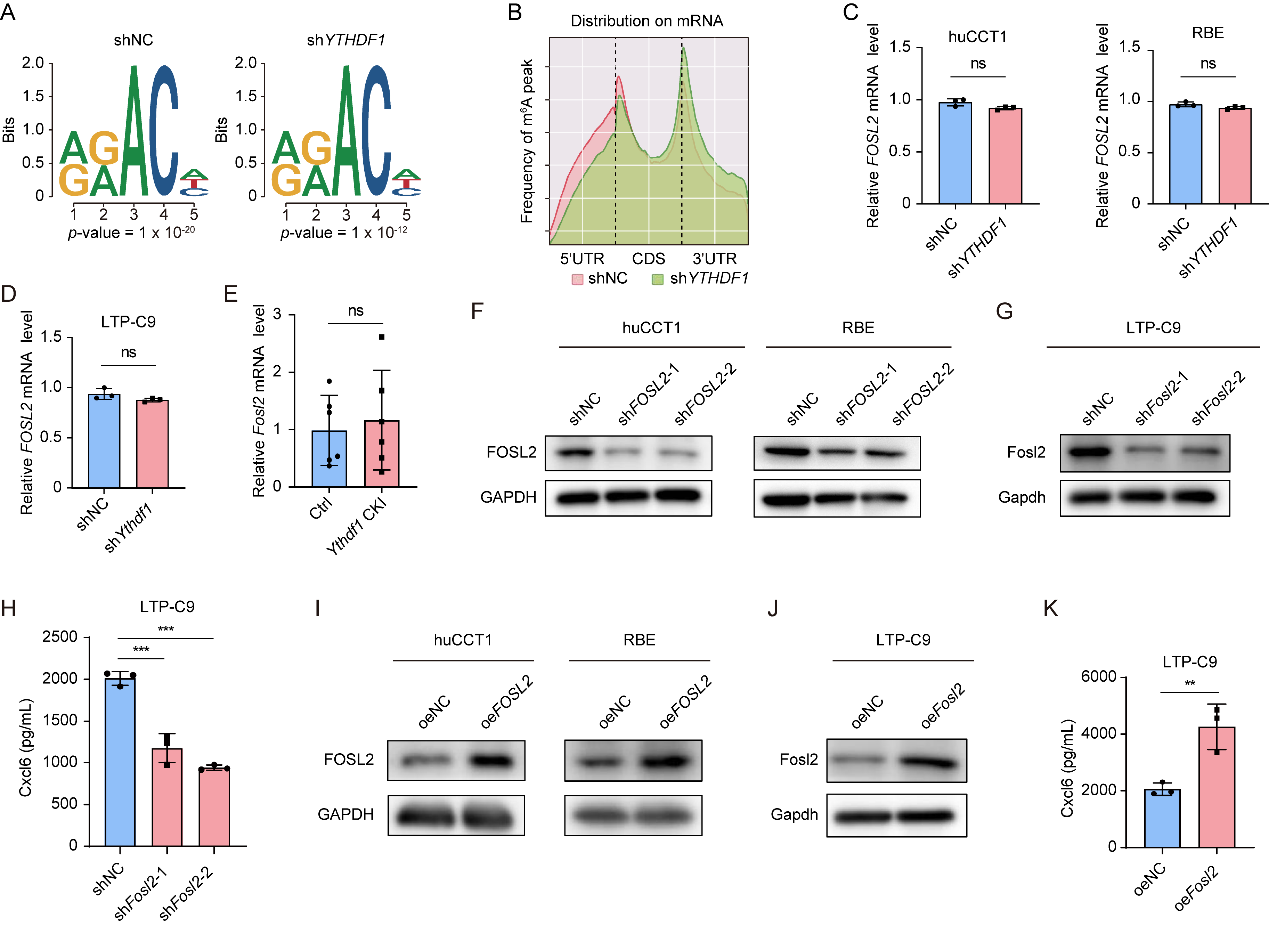


**Figure S8.** YTHDF1 promotes CXCL6 expression through FOSL2. A) Top enriched motifs from m^6^A peaks in huCCT1 shNC and sh*YTHDF1* cells identified by MeRIP-seq. B) Distribution of m^6^A peaks across 5’ untranslated region (UTR), coding sequences (CDS), and 3’ UTR of mRNA in huCCT1 shNC and sh*YTHDF1* cells by MeRIP-seq. C,D) RT-qPCR analysis of *FOSL2* mRNA level in *YTHDF1*-knockdown and control huCCT1 and RBE cells (C) and LTP-C9 cells (D). E) RT-qPCR showing *Fosl2* mRNA relative expression level in Ctrl and *Ythdf1* CKI ICC tumors. F,G) Western blot validated FOSL2-knockdown efficiency in huCCT1 and RBE cells (F) and LTP-C9 cells (G). H) ELISA showing Cxcl6 level in conditioned medium of shNC and sh*Fosl2* LTP-C9 cells. I,J) Western blot confirmed FOSL2 overexpression efficiency in huCCT1 and RBE cells (I) and LTP-C9 cells (J). K) ELISA analysis showing Cxcl6 level in conditioned medium of oeNC and oe*Fosl2* LTP-C9 cells. Data are presented as means ± SD, by Student’s t test (C-E, K) and by one-way anova (H); ns, no significance; **p* < 0.05; ***p* < 0.01; ****p* < 0.001.


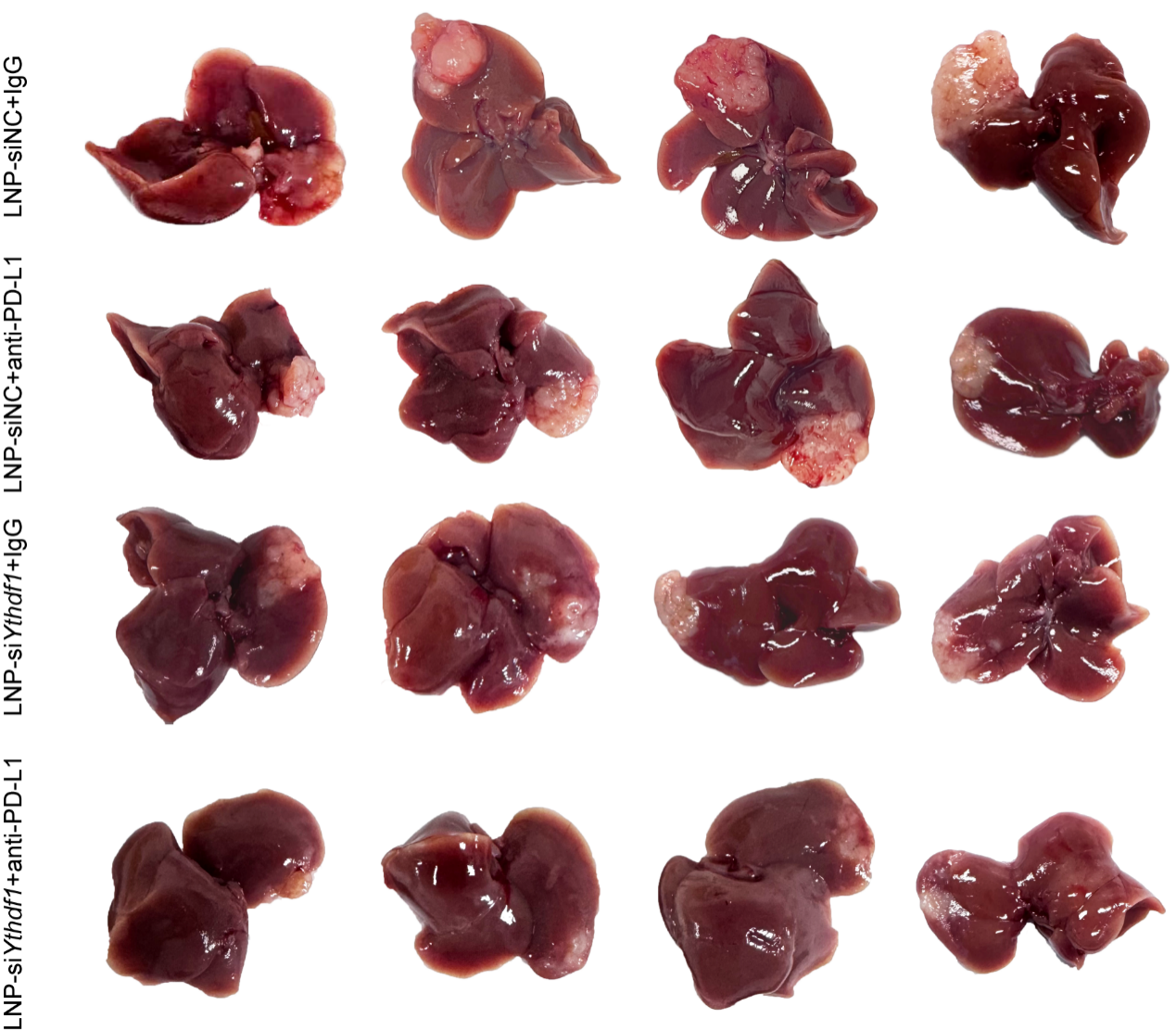


**Figure S9.** LNP-si*Ythdf1* in combination with anti-PD-L1 treatment reduces tumor burden. Gross liver pathological photographs of the indicated orthotopic tumors. Remaining tumor images from indicated orthotopic tumor, supplementing the representative images shown in Figure 5B.
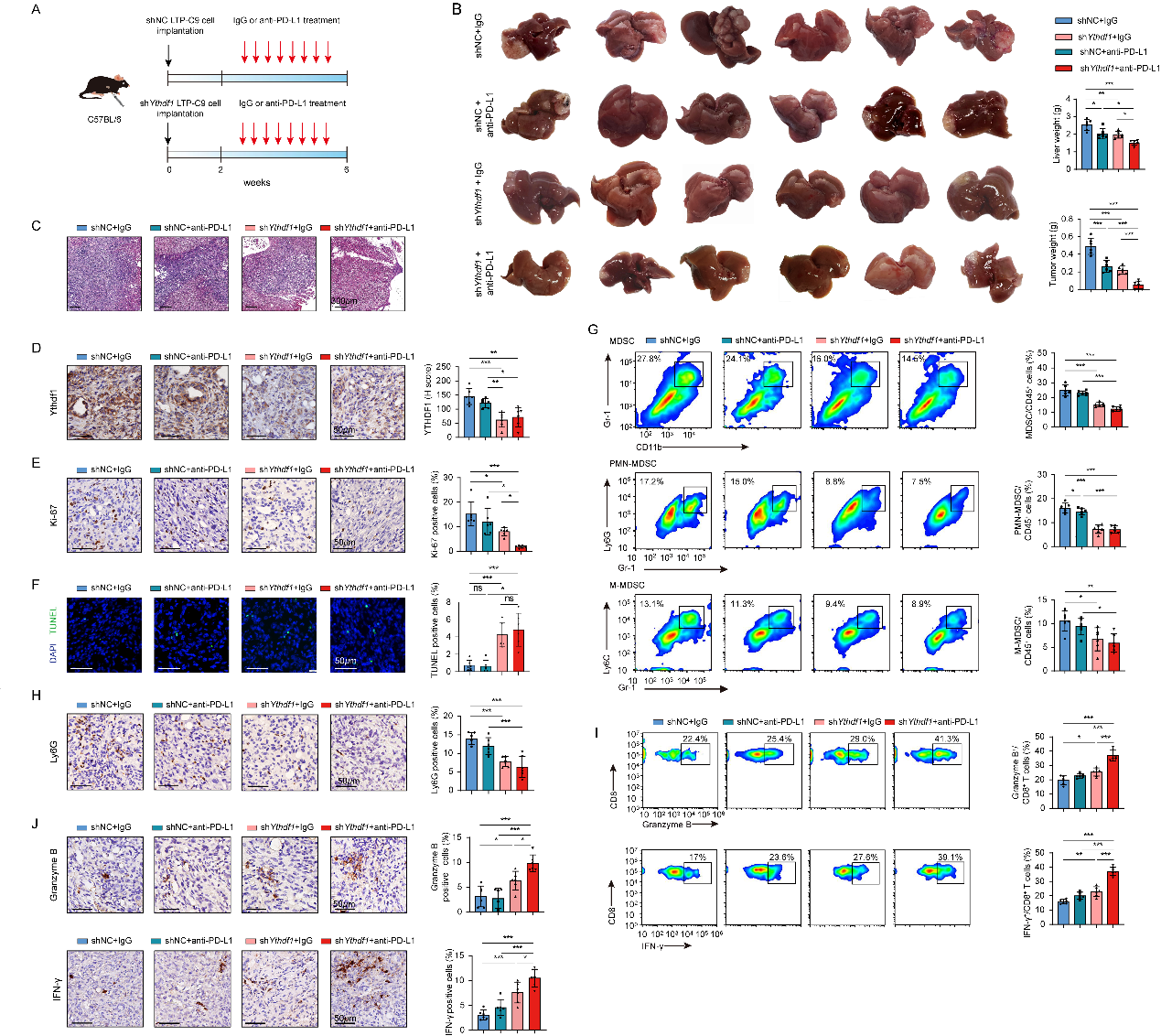


**Figure S10.** *Ythdf1*-knockdown improves the effect of anti-PD-L1 treatment by reduction of MDSCs and increase of functional CD8^+^ T cells in ICC orthotopic tumors. A) Schematic diagram of the orthotopic ICC model established by LTP-C9 tumor cell orthotopic implantation and subsequent anti-PD-L1 treatment. B) Gross liver pathological photographs of representative images and statistical analysis of ICC tumors of the orthotopic model treated with anti-PD-L1 (n = 6). C) H&E staining in the orthotopic model treated with anti-PD-L1 (n = 6). D,E) Representative IHC staining and quantification of YTHDF1 (D) and Ki-67 (E) in the indicated orthotopic tumors (n = 6). F) Representative TUNEL staining and quantification in the indicated orthotopic tumors (n = 6). G) Flow cytometry analysis of MDSCs (upper), PMN-MDSCs (median) and M-MDSCs (lower) in the indicated orthotopic tumors (n = 6). H) Representative IHC staining and quantification of Ly6G in the indicated orthotopic tumors (n = 6). I) Flow cytometry analysis of Granzyme B^+^ (upper) and IFN-γ^+^ (lower) CD8^+^ T cells in orthotopic tumors (n = 6). J) Representative IHC staining and quantification of Granzyme B (upper) and IFN-γ (lower) in orthotopic tumors (n = 6). Data are presented as means ± SD, by one-way anova (B, D-I); **p* < 0.05; ***p* < 0.01; ****p* < 0.001.
